# Impact of Arabinoxylan Consumption on Glycemic Control: A Systematic Review and Meta-Analysis of Preclinical and Clinical Studies

**DOI:** 10.3390/nu17172840

**Published:** 2025-08-31

**Authors:** Yujing Xu, Yuxin Liang, Jung Eun Kim

**Affiliations:** 1 Department of Food Science and Technology, National University of Singapore, Singapore 117543, Singapore; yujingxu@u.nus.edu (Y.X.); yuxinliang@u.nus.edu (Y.L.); 2 Bezos Centre for Sustainable Protein, National University of Singapore, Singapore 117542, Singapore

**Keywords:** cereal fiber, glycemic control, insulin sensitivity, type 2 diabetes mellitus

## Abstract

**Background/Objectives**: Arabinoxylan (AX) has shown potential benefits in glycemic control; however, findings remain inconclusive. This systematic review and meta-analysis aimed to assess the impact of AX intake on glycemic control in preclinical and clinical studies. **Methods**: A database search was conducted in PubMed, Embase, Cochrane Library, and CINAHL. A total of 133 studies were included for systematic review and extracted data from 46 clinical studies and 25 preclinical studies were further analyzed for meta-analysis. **Results**: The AX consumption improved overall postprandial glycemic control in clinical studies, as evidenced by reductions in glucose iAUC (SMD: −0.41; 95% CI: [−0.57, −0.25]), insulin iAUC (SMD: −0.28; 95% CI: [−0.44, −0.12]), glucose iPeak (SMD: −0.52; 95% CI: [−0.80, −0.25]), and insulin iPeak (SMD: −0.24; 95% CI: [−0.41, −0.06]) compared to the control. For chronic glycemic control, fasting glucose (Hedges’ g: −1.18; 95% CI: [−1.56, −0.80]), insulin (Hedges’ g: −1.07; 95% CI: [−1.92, −0.23]), HbA1c (Hedges’ g: −2.93; 95% CI: [−5.48, −0.38]), and HOMA-IR (Hedges’ g: −2.44; 95% CI: [−3.66, −1.22]) reduced in preclinical studies, while improvements were limited to fasting glucose (MD: −0.10; 95% CI: [−0.16, −0.03]) in clinical studies. Subgroup analyses revealed that AX exerted a greater glycemic-lowering effect in metabolically impaired animals and individuals compared to healthy counterparts. Furthermore, extracted AX was found to be more effective than intrinsic AX in optimizing glycemic control. **Conclusions**: The consumption of AX improves glycemic control, particularly in metabolically impaired animals and human participants. Moreover, the benefit appears more pronounced with extract AX interventions.

## 1. Introduction

Impaired glycemic control is characterized by an elevated postprandial glycemic response and chronic glycemia [1,2], and numerous studies have identified impaired glycemic control as a significant risk factor for the development and progression of chronic diseases, such as type 2 diabetes mellitus (T2DM) and cardiovascular diseases (CVDs) [3,4,5,6,7]. Moreover, in individuals who are diagnosed with T2DM, poor glycemic control further contributes to the onset of diabetes-related complications, leading to increased morbidity and mortality [2]. Observational studies reported that the prevalence of impaired glycemic control ranges from 50.1% to 91.8%, posing a significant public health challenge, and thus an effective strategy for optimizing glycemic control is urgently needed [8].

Dietary modifications have been widely adopted as a non-pharmacological approach to regulating glycemic control [9], and higher dietary fiber intake has been particularly highlighted. Dietary fiber cannot be hydrolyzed by enzymes in the human digestive tract. Soluble dietary fiber interacts with chyme to increase its viscosity, which slows gastric emptying and subsequently attenuates the postprandial glycemic response [10]. Although the primary function of insoluble dietary fiber is to increase fecal bulk and decrease intestinal transit time, it may also help regulate glycemic response by reducing starch-digesting enzyme activity, thereby slowing starch digestion and enhancing glycemic control [11]. Additionally, the fermentable dietary fiber can be utilized by gut microbiota, leading to the production of short-chain fatty acids (SCFAs), which play a crucial role in glucose homeostasis. Notably, results from prospective cohort studies suggest that higher cereal dietary fiber intake is negatively associated with T2DM risk [12,13], and this may be because of the greater efficacy of cereal dietary fiber in enhancing insulin sensitivity [14,15,16].

Arabinoxylan (AX) is a common cereal dietary fiber that can be widely found in the bran layer of cereals, such as wheat bran, rice bran, and barley bran [17]. Some clinical studies have reported that the incorporation of AX or AX-containing cereal into test meals can improve the postprandial glycemic response in individuals with metabolic syndrome or T2DM [18,19]. Furthermore, long-term consumption of AX-containing foods has been shown to lower glucose, insulin, and hemoglobin A1c (HbA1c) concentrations and improve insulin sensitivity in preclinical and clinical studies [20,21,22]. Moreover, a recent systematic review and meta-analysis revealed that AX can modulate gut microbiota abundance and promote SCFAs production, which may further regulate glucose metabolism [23]. Collectively, these findings suggest that consumption of AX or AX-containing cereal food may effectively improve glycemic control. However, the findings across the literature remain inconsistent, and these inconsistencies may be attributed to differences in the metabolic health status of the participants. Moreover, the source of AX may be another plausible reason. Intrinsic AX, naturally occurring in the cereal cell wall, is crosslinked with other wall components, which limits its solubility and fermentability [24]. In contrast, the extraction process can modify its structural characteristics, such as degree of branching and molecular weight, thereby increasing solubility and potentially altering its physiological effects [24].

Therefore, the aim of the present systematic review and meta-analysis was to evaluate the effectiveness of AX-based interventions in postprandial and chronic glycemic control in both preclinical and clinical studies. Subgroup analyses were further performed to compare the impact of AX across different metabolic health status and between various sources of AX.

## 2. Materials and Methods

This systematic review, meta-analysis, and meta-regression were conducted in accordance with the Preferred Reporting Items for Systematic Reviews and Meta-Analyses (PRISMA) guidelines. The protocol of the current study was registered at the International Prospective Register of Systematic Reviews as CRD42024591193.

### 2.1. Search Strategy and Selection Criteria

The PICOS (participant, intervention, comparison, outcome, and study design) statement for the current study is summarized in Table 1. A computerized search for the available literature was independently conducted by a primary reviewer (Y. X.) and a secondary reviewer (Y. L.) across four databases: PubMed, Embase, Cochrane Library, and CINAHL. The initial search was performed in March 2024 and updated in February 2025. Details of the search terms and filters applied in each database can be found in Supplemental Table S1. Relevant studies were included according to the following inclusion criteria: (1) published in English; (2) randomized controlled trials involving adults (≥19 years) or animals; (3) compared either the effect of AX or the effect of higher dosage of AX; (4) reported at least one outcome of primary interest—postprandial glucose response, postprandial insulin response, fasting glucose, fasting insulin, homeostatic model assessment of insulin resistance (HOMA-IR), or HbA1c.

Firstly, all studies identified from the selected databases were imported into EndNote X9 for duplicate removal. The remaining studies were then screened based on their title and abstract to assess their potential relevance. Studies deemed relevant were retrieved in full text for further eligibility evaluation. The entire screening process was conducted independently by the primary (Y. X.) and secondary (Y. L.) reviewers. Any discrepancies in the reviewers’ decisions were resolved through consultation with the third reviewer (J. E. K.).

### 2.2. Data Extraction

For the eligible studies, the following details were extracted by primary (X. Y.) and secondary (Y. L.) reviewers independently: study title, author, publication year, study design, the characteristics of animal models and human participants (population/animal, gender, metabolic health status, mean age, and mean BMI), sample size, intervention duration, intervention dosage, and type of AX. The type of AX was categorized into extracted AX and intrinsic AX. Extracted AX was defined as AX that has been isolated and purified from its original food matrix, irrespective of its subsequent incorporation into other food systems for intervention. In contrast, intrinsic AX was defined as AX naturally occurring in cereals, retaining its original structural composition and interaction with the food matrix, such as wheat bran, barley bran, and rye bran. For postprandial glycemic response, the area under the curve (AUC) and incremental area under the curve (iAUC) for glucose and insulin, as well as the peak concentration (Peak) and incremental peak concentration (iPeak), were collected. The 2 h AUC and iAUC were preferred. If a study did not report the 2 h values, the closest available time point was used. For chronic glycemic control, fasting glucose, fasting insulin, HbA1c, and HOMA-IR were collected. Secondary outcomes, including BMI, blood pressure, total cholesterol (TC), low-density lipoprotein cholesterol (LDL-C), HDL-C, and triglycerides (TG), were collected only if the study reported at least one primary outcome. When relevant data were presented in figures, PlotDigitizer (Free Online App) was used to extract mean and standard deviation values. Corresponding authors were contacted when there was missing data.

The studies with a crossover design were analyzed as a parallel design, considering the full number of participants representing both the AX consumption group and the control group. Studies with multiple intervention arms were treated as separate groups, with each intervention analyzed independently against the control.

### 2.3. Risk of Bias Assessment

The quality assessment of the studies included in the meta-analysis was independently performed by two reviewers (Y. X. and Y. L.). For preclinical studies, a modified SYRCLE’s risk of bias tool was applied, assessing bias from the randomization, missing data, selective reporting, and other potential sources [25]. For clinical studies, a modified version of the Cochrane Risk of Bias tool (Version 2) was employed [26]. This assessment covered the following domains: bias arising from the randomization process, bias due to deviations from the intended intervention, bias due to missing outcome data, bias in measurement of the outcome, and bias in selection of the reported result. In addition, for the clinical studies with a crossover design, an additional domain, bias arising from period and carryover effects, was also evaluated. Any discrepancies in the reviewers’ decisions were resolved through discussion with the third reviewer (J. E. K.). Additionally, the risk of bias in the selected studies was also examined via visual inspection of the funnel plot and Eggar’s regression test.

### 2.4. Data Synthesis and Statistical Analysis

The unit of glucose AUC and iAUC was standardized to mmol·min/L, while the unit of insulin AUC and iAUC was standardized to pmol·min/L. Fasting glucose, insulin, and lipid–lipoprotein concentrations (TC, LDL-C, HDL-C, and TG) were standardized to SI units, mmol/L, pmol/L, and mmol/L, respectively.

When studies reported mean and standard error or median and interquartile range values, it was converted into mean and standard deviation. The standardized mean difference (SMD) and corresponding standard error were calculated to determine the effect size of the AUC, iAUC, Peak, and iPeak of glucose and insulin in clinical studies. For preclinical studies, the adjusted standardized mean difference (Hedges’ g) and standard error were calculated by adjusting for the small sample size constant. Moreover, the absolute mean difference (MD) and standard error were calculated in clinical studies to determine the effect size of chronic glycemic control indicators. All calculations followed the Cochrane Handbook [27].

R was used for the meta-analysis of the extracted data. The metagen function in the metafor R (4.4.0) package was used to determine the pooled effect of AX consumption on glycemic control in preclinical and clinical studies. The pooled effect was expressed by MDs with their 95% CIs. Heterogeneity was assessed using the *I*^2^ statistic, with *I*^2^ > 50% indicating significant heterogeneity. When substantial heterogeneity was present (*I*^2^ > 50%), a random-effects model was applied to evaluate the pooled effect. Conversely, a fixed-effects model was used when heterogeneity was low (*I*^2^ ≤ 50%). To explore the source of heterogeneity, the sensitivity test with the leave-one-out function in metafor R package was conducted. Moreover, subgroup analyses were performed by segregation comparison based on the participants’ metabolic health status (metabolically impaired vs. metabolically healthy) and the type of AX (extracted AX vs. intrinsic AX).

## 3. Results

### 3.1. Search Results

**Figure 1:**
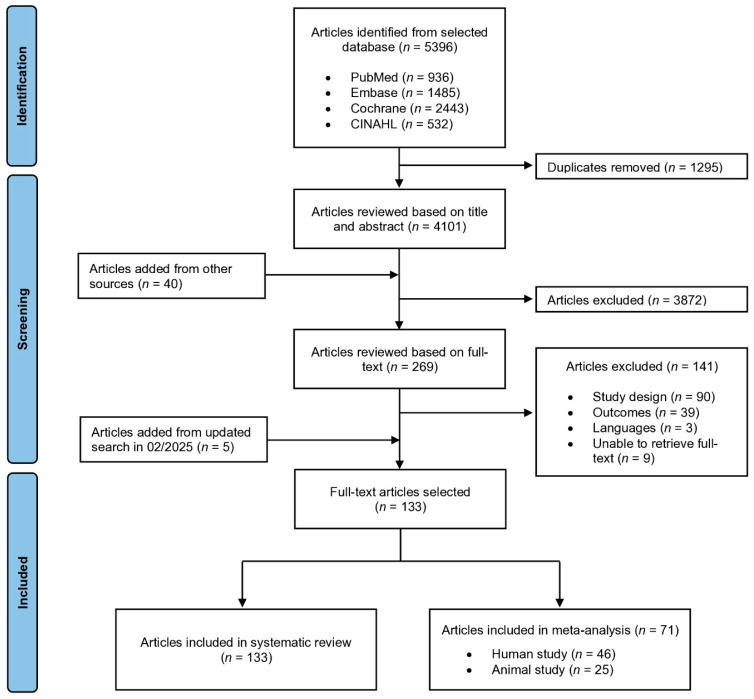
PRISMA flow chart for the systematic review and meta-analysis.

The search flow and data extraction process are illustrated in Figure 1. A total of 5396 studies were initially identified from the selected databases. After removing duplicates (*n* = 1295) and screening titles and abstracts based on the inclusion criteria, along with additional articles identified from other sources such as Web of Science and Elsevier (*n* = 40), 269 articles were selected for full-text review. An additional five articles were added after the updated search in February 2025. Following full-text screening, 141 studies were excluded for the following reasons: ineligible study design with irrelevant interventions or control groups (*n* = 90), did not report the primary outcomes (*n* = 39), not published in English (*n* = 3), and unable to be retrieved in full text (*n* = 9). Finally, 133 studies were included in the systematic review, and of these, 71 studies were eligible for meta-analysis, comprising 46 clinical studies and 25 preclinical studies.

### 3.2. Study Characteristics

The detailed characteristics of all included studies are summarized in Supplemental Tables S2–S4. For the analysis of the postprandial glycemic response in humans (Supplemental Table S2), 26 studies (48 comparisons) involving 758 participants were included [18,19,28,29,30,31,32,33,34,35,36,37,38,39,40,41,42,43,44,45,46,47,48,49,50,51,52], among them, 312 participants with metabolic impairments, including metabolic syndrome, impaired glucose tolerance, and obesity. Nine comparisons used extracted AX at doses ranging from 6 g to 15 g, while the remaining comparisons used intrinsic AX, primarily derived from wheat bran and rye.

With respect to chronic glycemic control in humans (Supplemental Table S3), 21 studies with a total of 1017 participants were included [20,21,36,53,54,55,56,57,58,59,60,61,62,63,64,65,66,67,68,69], of whom 550 were metabolically impaired. Among the included studies, 10 studies were with a crossover design, while the remaining utilized a parallel design. Moreover, a total of 23 comparisons were analyzed, with 7 comparisons administered extracted AX in doses ranging from 2.2 g to 15 g, and 16 comparisons consumed intrinsic AX. The main sources of intrinsic AX were wheat bran and rye bran. The intervention duration varied from 1 to 12 weeks, with eight studies lasting 12 weeks.

Regarding chronic glycemic control in preclinical studies (Supplemental Table S4), 27 animal studies with 50 comparisons were included [22,70,71,72,73,74,75,76,77,78,79,80,81,82,83,84,85,86,87,88,89,90,91,92,93]. Among these, 17 studies investigated potential underlying mechanisms of glycemic control, such as the expression of genes involved in glucose metabolism. Of the 50 comparisons, 26 involved metabolically impaired animal models, including T2DM, hypercholesterolemia, and obesity. Furthermore, 17 comparisons used extracted AX, while 33 comparisons used intrinsic AX as the intervention. The primary sources of intrinsic AX were wheat bran and rice bran and study durations ranged from 2 to 18 weeks.

### 3.3. Effects of AX on Glycemic Control

#### 3.3.1. Postprandial Glycemic Response

The meta-analysis showed that consumption of AX significantly improved the postprandial glycemic response across multiple indicators in clinical studies (Table 2). Significant reductions were observed in glucose iAUC (SMD: −0.41; 95% CI: [−0.57; −0.25]) and insulin iAUC (SMD: −0.28; 95% CI: [−0.44; −0.12]). Similarly, glucose iPeak (SMD: −0.52; 95% CI: [−0.80; −0.25]) and insulin iPeak (SMD: −0.24; 95% CI: [−0.41; −0.06]) were also reduced after the AX consumption. Additionally, favorable changes were observed for the AUC and Peak of glucose and insulin as well.

#### 3.3.2. Chronic Glycemic Control

**Figure 2:**
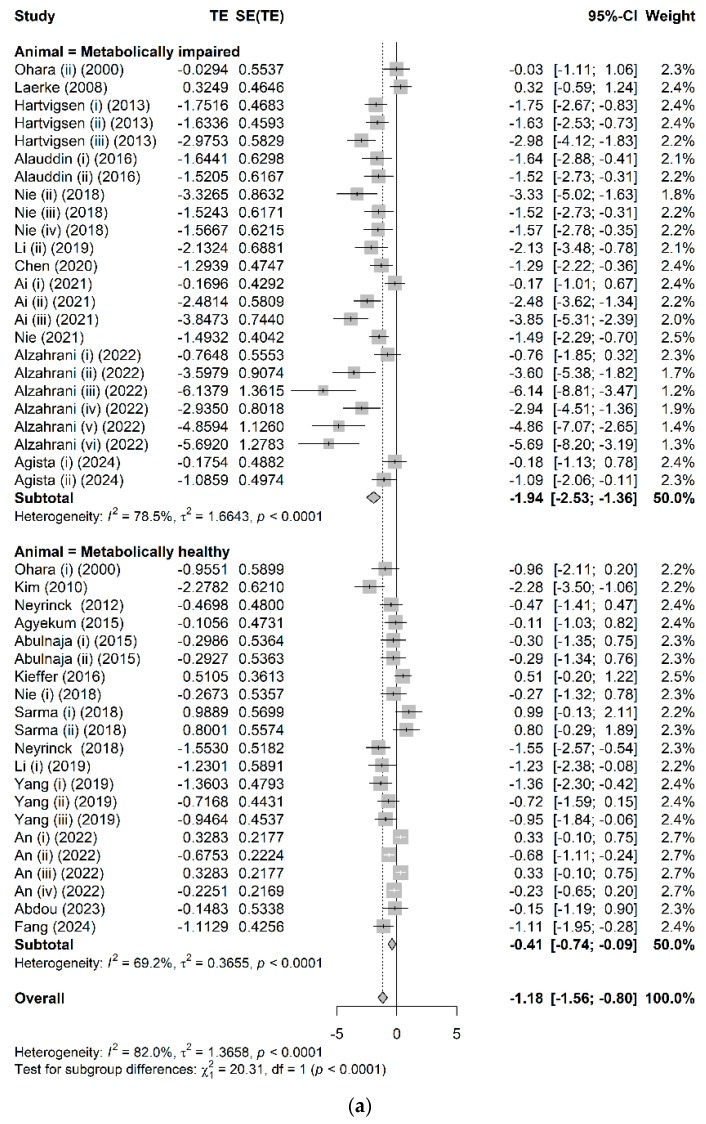

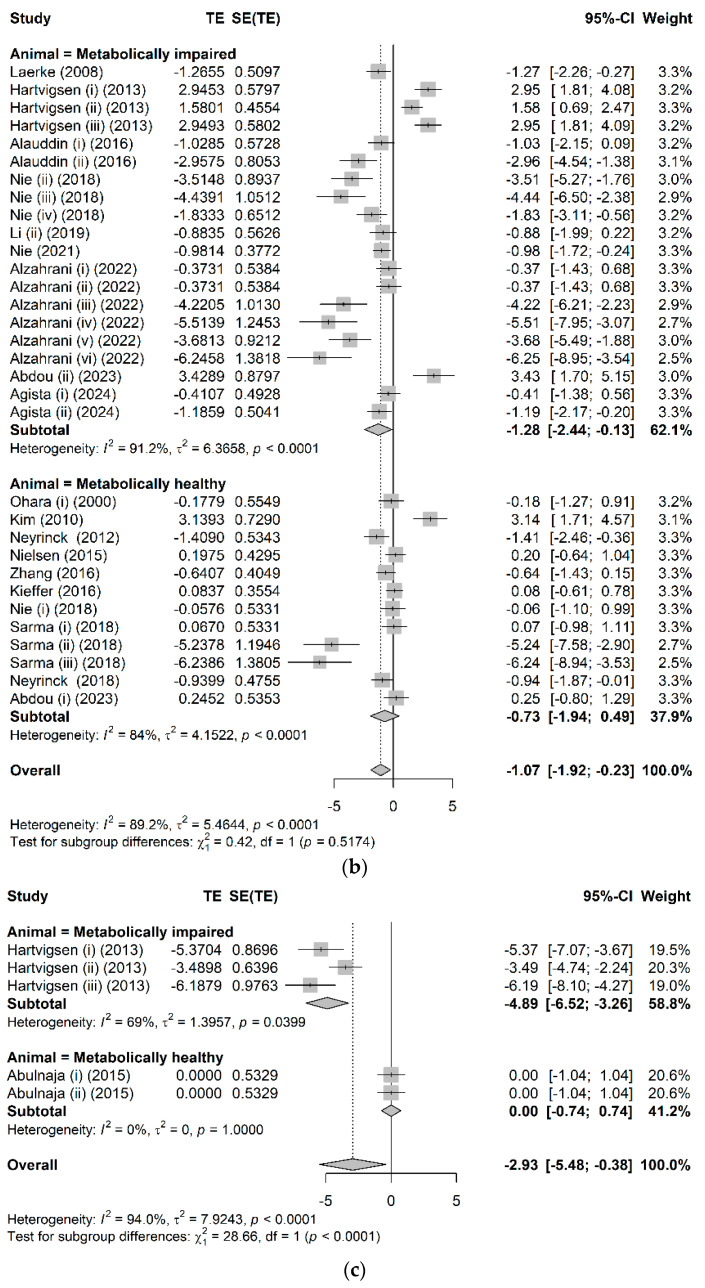

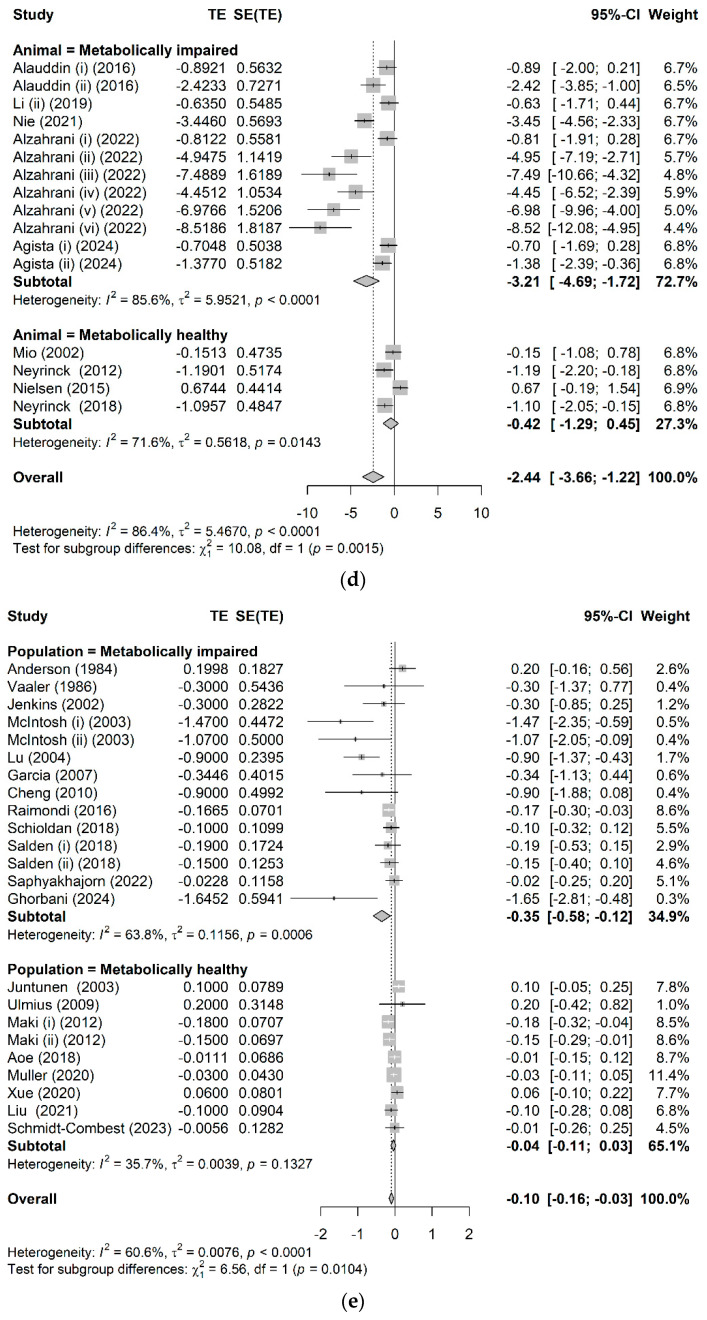

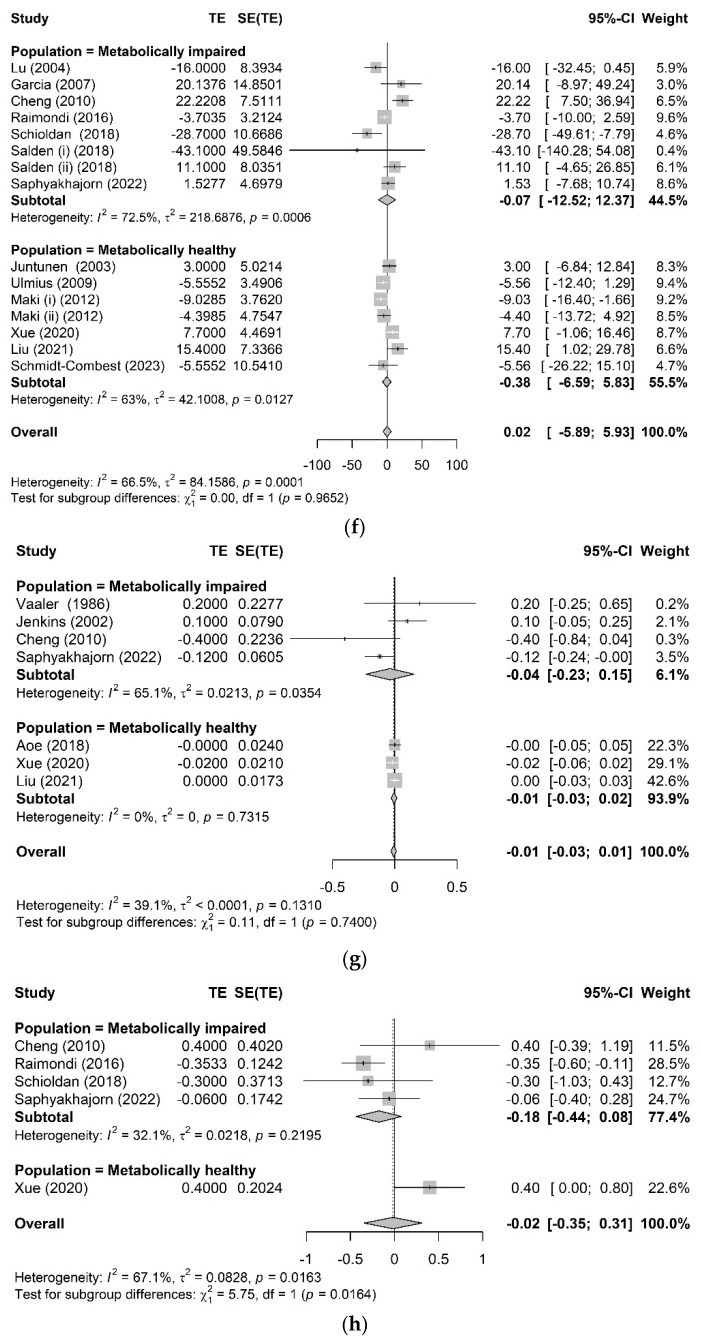
Forest plots of meta-analysis evaluating the effect of AX consumption on chronic glycemic control, with subgroup analysis based on the metabolic health status of study cohorts. The effects of AX consumption on (**a**) Fasting glucose; (**b**) Fasting insulin; (**c**) HbA1c; and (**d**) HOMA-IR in preclinical studies; and (**e**) Fasting glucose; (**f**) Fasting insulin; (**g**) HbA1c; and (**h**) HOMA-IR in clinical studies. ([20,21,22,36,53,54,55,56,57,58,59,60,61,62,63,64,65,66,67,68,69,70,71,72,73,74,75,76,77,78,79,80,81,82,83,84,85,86,87,88,89,90,91,92,93]).

The effects of AX consumption on chronic glycemic control-related biomarkers, including fasting glucose, fasting insulin, HbA1c, and HOMA-IR, are shown in Figure 2. In preclinical studies, AX consumption resulted in overall improvements across all glycemic control-related biomarkers (Figure 2a–d). The mechanisms underlying these effects, as explored in preclinical studies, include modulation of gene expressions involved in glucose metabolism, such as AMP-activated protein kinase (AMPK); favorable alterations in the gut microbiota composition; and enhanced production of SCFAs (Supplemental Table S4). In clinical studies, although there was a significant reduction in fasting glucose concentration after AX consumption (MD: −0.10; 95% CI: [−0.16; −0.03]) (Figure 2e), no effects were observed in other glycemic control-related biomarkers (Figure 2f–h).

According to subgroup analysis based on metabolic health status, both animal models and human participants with metabolic impairments exhibited a more pronounced response to AX intervention compared to their metabolically healthy counterparts. In preclinical studies, significant reductions were noted in fasting insulin (Hedges’ g: −1.28; 95% CI: [−2.44, −0.13]), HbA1c (Hedges’ g: −4.89; 95% CI: [−6.52, −3.26]) concentrations, and HOMA-IR (Hedges’ g: −3.21; 95% CI: [−4.69, −1.72]), exclusively in metabolically impaired animal models following AX intervention. Similarly, in clinical trials, a significant reduction in the fasting glucose concentration (MD: −0.35; 95% CI: [−0.58, −0.12]) was observed in metabolically impaired participants, whereas no improvement was found in metabolically healthy participants (MD: −0.04; 95% CI: [−0.11, 0.03]).

Furthermore, subgroup analysis based on the source of AX (Supplemental Table S5) revealed that extracted AX has greater efficacy in glycemic control compared to intrinsic AX. In both preclinical and clinical studies, the fasting insulin concentration was significantly reduced with extracted AX (preclinical: Hedges’ g: −1.14; 95% CI: [−2.25, −0.02]; clinical: MD: −0.15; 95% CI: [−0.24, −0.06]) but not with intrinsic AX (preclinical: Hedges’ g: −1.01; 95% CI: [−2.36, 0.35]; clinical: MD: −0.05; 95% CI: [−0.13, 0.03]). No significant differences were observed between extracted and intrinsic AX for other glycemic control-related biomarkers.

#### 3.3.3. Other Metabolic Health-Related Biomarkers

The effects of AX consumption on other metabolic health-related biomarkers are summarized in Supplemental Table S6. In preclinical studies, AX consumption significantly improved lipid–lipoprotein profiles, and further subgroup analysis revealed that the HDL-C concentration increased significantly in metabolically impaired animal models (Hedges’ g: 1.21; 95% CI: [0.31, 2.11]) but not in metabolically healthy models (Hedges’ g: 0.06; 95% CI: [−0.28, 0.41]). In clinical studies, no significant improvements were observed in BMI, blood pressure, and lipid–lipoprotein profiles, except for an increase in the HDL-C concentration (MD: 0.02; 95% CI: [0.00, 0.05]).

### 3.4. Publication Bias and Sensitivity Test

The results of the sensitivity test for the primary outcomes are presented in Supplemental Tables S7–S22. The omission of individual studies did not lead to any significant changes in the results, indicating that the findings of this meta-analysis are generally robust.

The risk of bias assessment of individual studies is detailed in Supplemental Figures S1 and S2. In preclinical studies, 10 were judged as having some concerns due to insufficient information on randomization, and 3 were rated as high risk due to unexplained missing outcome data (*n* = 2) and selective reporting (*n* = 1). Overall, 10 preclinical studies were considered to have a low risk of bias across the four assessed domains. Among clinical studies, five studies were assessed as high risk because of improper randomization (*n* = 2), insufficient washout periods (*n* = 2), and missing outcome data (*n* = 1). In total, 13 studies were considered to have a low risk of bias across all assessed domains. Furthermore, the funnel plot and Egger’s test results for outcomes with more than 10 comparisons are presented in Supplemental Figures S3–S5. Evidence of significant publication bias was observed for postprandial glucose AUC, postprandial glucose Peak, postprandial glucose iPeak, fasting glucose in preclinical studies, fasting insulin in preclinical studies, HOMA-IR in preclinical studies, and fasting glucose and fasting insulin in clinical studies (Egger’s test *p* < 0.05).

## 4. Discussion

AX is a cereal dietary fiber that has shown potential benefits in glycemic control, which will subsequently reduce the risk of insulin resistance and T2DM [94]. Results from the current systematic review and meta-analysis confirm that AX consumption can improve both postprandial and chronic glycemic control-related biomarkers. Moreover, subgroup analyses reveal that the AX consumption had a more pronounced effect on glycemic control in metabolically impaired animal models and human participants. In addition, extracted AX was also found to be more effective than intrinsic AX in optimizing glycemic control.

Postprandial hyperglycemia is increasingly being recognized as a relevant risk factor of chronic diseases, such as T2DM [95]; thus, optimizing postprandial hyperglycemia is crucial for the prevention and management of T2DM. Our results showed that AX consumption favorably improved postprandial glycemic control, and this finding is consistent with a previous meta-analysis, which reported that whole grain rice consumption significantly attenuates the postprandial glucose response [96]. The underlying mechanisms are well studied: Firstly, AX can inhibit the activity of α-amylase and α-glucosidase, which limit the breakdown of available carbohydrates into absorbable monosaccharides, as reported in previous in vitro studies [97,98]. Moreover, AX can increase the viscosity of chyme, which, in turn, delays gastric emptying and the absorption of glucose in the small intestine [10]. For example, in a study conducted in ileal-cannulated pigs, the viscosity of the ileal digesta was found to be three times higher following the consumption of AX-containing bread compared to white bread [99].

Regarding chronic glycemic control, the reduction of the glucose concentration was observed in both preclinical and clinical studies following AX intake. One potential mechanism underlying the observed improvement is that AX consumption increases the production of SCFAs [61,62,100,101]. SCFAs play a crucial role in regulating glucose metabolism by stimulating the secretion of gut hormones, such as glucagon-like peptide-1 and peptide tyrosine tyrosine, and by modulating the expression of glucose transporter-4 in skeletal muscle [102]. Additionally, the intake of AX can improve chronic glycemic control by enhancing insulin sensitivity and reducing insulin resistance, which ultimately promotes glucose uptake into peripheral tissues (muscle and adipose) and suppresses hepatic glucose production [103]. A previous study demonstrated that feeding Zucker diabetic fatty rats for 7 weeks with AX-containing bread significantly altered the expression of insulin sensitivity-related genes, including the upregulation of adipose adiponectin receptors-1 and AMPK [74]. These upregulations promote the glucose uptake into adipocytes, as well as glycogenesis, thereby improving insulin sensitivity [104,105]. 12α-hydroxylated bile acids have been proven to trigger hepatic steatosis and ultimately cause insulin resistance [106,107]. A recent study observed a decrease in serum 12α-hydroxylated bile acids concentrations in streptozotocin-induced T2DM rats after 4 weeks of supplementation with 100 or 200 mg/kg AX, suggesting an improvement in insulin resistance [108]. Gut microbiota modulation represents another potential mechanism. AX intake has been shown to increase the abundance of butyrate-producing bacteria, such as *Bifidobacterium*, while reducing opportunistic pathogens like Proteobacteria, which collectively enhance insulin sensitivity [108,109]. Furthermore, the Firmicutes/Bacteroidetes ratio is a microbiome marker closely linked to the development of insulin resistance [110]. AX consumption has been associated with a decrease in Firmicutes and an increase in Bacteroidetes, contributing to improved insulin resistance [111,112]. Despite these positive effects on glucose and insulin sensitivity, the indicator for long-term glycemic control, HbA1c, remains unchanged in clinical studies. The absence of an effect on HbA1c is likely due to the source of AX, as all studies included in this meta-analysis used intrinsic AX, which is less soluble. Results of subgroup analysis from Mao’s study revealed that soluble fiber was more effective in reducing HbA1c compared to the dietary fiber naturally occurring in food [113].

Results of subgroup analysis based on metabolic health status suggest that the glycemic-controlling effect of AX is more distinct in animals and individuals with metabolically impaired status than in metabolically healthy counterparts. This may be attributed to the fact that those with metabolic impairments exhibit impaired glycemic control, making them more responsive to AX intervention [1]. In Nie’s study, both healthy rats and streptozotocin-induced diabetic rats were administered 200 mg/kg body weight of AX for 4 weeks, and the glucose concentration was significantly lower in diabetic rats when they received the AX intervention compared to the diabetic control, while no difference was observed in the healthy rats [81]. Similarly, when barley insoluble fiber (primarily AX) was administered to both healthy and diabetic rats for 4 weeks, a reduction in the glucose concentration was only observed in diabetic rats [83]. Consistently, a recent meta-analysis also revealed that the effect on fasting glucose was only confined to participants with metabolic impairments, rather than to healthy populations, following the consumption of bread enriched with dietary fiber or whole grains [114]. When considering the source of AX, extracted AX is more effective in glycemic control compared to intrinsic AX. The intrinsic AX occurring in the cereal bran is mostly crosslinked with other cell wall components to form a structural network that limits its solubility [115]. However, chemical and enzymatic extractions can break the hydrogen and covalent bonds within the cell wall matrix and the linkages between monomer units in the AX molecule. These changes affect the structure and molecular weight of AX, subsequently altering its functional properties, such as solubility, viscosity, and fermentability [115,116]. As a result, extracted AX may exhibit a superior effect on managing glucose homeostasis by more effectively delaying glucose absorption and being more readily utilized by gut microbiota. Supporting this, a recent meta-analysis found that extracted AX consumption more effectively increases the abundance of *Bifidobacterium*, a SCFAs-producing bacterium associated with improved glycemic control [23].

Besides the findings in primary outcomes, we also found that AX consumption can enhance lipid–lipoprotein metabolism in animal models. The hypolipidemic effect of AX is well recognized in animal studies, where it has been shown to inhibit de novo lipogenesis, promote cholesterol catabolism, and enhance lipid excretion [117,118]. Additionally, these improvements in the lipid–lipoprotein profile may indirectly support glycemic control by reducing ectopic fat accumulation and lipotoxicity, thereby enhancing insulin sensitivity in skeletal muscle [119]. In clinical studies, albeit a moderate increase in HDL-C concentration was noted, overall, there were no beneficial effects on lipid–lipoprotein profile regulation. This may be due to the relatively low viscosity of AX compared with gel-forming fibers, such as β-glucan, which reduce lipid absorption in the small intestine [99,120]. Supporting this, a network meta-analysis by Juhász, et al. [121] indicated AX had a lower efficacy for improving the lipid–lipoprotein profile in human studies, based on ranking among different types of dietary fiber. Additionally, AX may inhibit the activity of cholesterol 7α-hydroxylase, reduce cholesterol catabolism, and limit its lipid-lowering effect [122]. However, the results should be taken with caution, as they are the secondary outcome of the current study with relatively high heterogeneity. Future studies focusing primarily on lipid metabolism are needed to provide more robust conclusions.

It is noteworthy that discrepancies exist between the results of preclinical and clinical studies in both glycemic control and lipid metabolism, and this is likely attributed to the relatively higher AX dosage in preclinical studies. Several animal studies that tested a range of doses demonstrated greater metabolic improvements with higher AX dosages, suggesting a potential dose–response relationship [76,82,84,88]. In our meta-analysis, the median dosage of AX intake in preclinical studies is 0.5 mg/kg of body weight. When translating this dosage to a 60 kg human participant, it would correspond to approximately 30 g of AX, whereas the highest dosage supplemented in human studies is only 16 g. Additionally, preclinical studies are conducted under highly controlled conditions, while clinical studies face greater variability due to diverse demographics, diets, and lifestyles, making it more challenging to detect consistent effects.

The main strength of this study lies in the systematic evaluation of the impact of AX intake on a broad range of glycemic control-related biomarkers, including both postprandial and chronic measures, which offers a more comprehensive understanding of AX’s glycemic regulatory effects. Furthermore, we performed subgroup analyses based on the metabolic health status and AX sources that provide distinct effects on glycemic control. Lastly, by including preclinical studies, our study enables the exploration of potential mechanisms underlying the long-term effects of AX on glycemic control. However, several limitations are evident in this study. First, regarding the effect of AX on postprandial glycemic control, the available carbohydrate content of the test meals was not matched in all studies, which may introduce potential confounders into the results. Second, our analysis included interventions involving both AX supplementation and AX-containing foods. While this enhances the generalizability of the findings, it may also introduce heterogeneity, as the food matrix and other nutrients present in foods could potentially confound the interpretation of AX’s isolated impact. Additionally, although we compared AX from different sources (extracted vs. intrinsic), other physicochemical properties, such as molecular weight and solubility, may also influence the results.

## 5. Conclusions

In conclusion, our findings reveal that the consumption of AX improved both postprandial and chronic glycemic control, particularly in the metabolically impaired population. Moreover, taking the extract AX tends to offer more beneficial effects on glycemic control than taking the intrinsic form. These results collectively support the potential of AX as a functional ingredient in dietary strategies aimed at optimizing glycemic control and ultimately reducing the risk of chronic diseases.

## Figures and Tables

**Table 1:** PICOS criteria for inclusion of studies.

Parameter	Description
Population	Animals; Human subjects (mean age ≥ 19 years).
Intervention	Consuming arabinoxylan or arabinoxylan-containing cereal food; Consuming higher dosages of arabinoxylan or arabinoxylan-containing cereal food.
Comparison	Not consuming arabinoxylan or arabinoxylan-containing cereal food, or consuming placebo; Consuming lower dosages of arabinoxylan or arabinoxylan-containing cereal food.
Outcomes	Primary outcome: postprandial glycemic control-related biomarkers (postprandial glucose AUC, iAUC, Peak, iPeak, and postprandial insulin AUC, iAUC, Peak, iPeak); chronic glycemic control-related biomarkers (fasting glucose, fasting insulin, HbA1c, and HOMA-IR).
Study Design	Animal studies; Randomized controlled trials.

AUC: area under the curve; iAUC: incremental area under the curve; Peak: peak concentration; iPeak: incremental peak concentration; HbA1c: hemoglobin A1c; HOMA-IR: homeostatic model assessment of insulin resistance.

**Table 2:** The effects of AX consumption on postprandial glycemic response.

Outcomes	SMD	95% CI	*I*^2^ (%)	τ^2^	Model	Comparisons Included (*n*)
Glucose AUC	−0.34	[−0.58; −0.10]	51%	0.14	Random	19
Glucose iAUC	−0.41	[−0.57; −0.25]	0%	0	Fixed	18
Insulin AUC	−0.42	[−0.67; −0.17]	0%	0	Fixed	9
Insulin iAUC	−0.28	[−0.44; −0.12]	0%	0	Fixed	18
Glucose Peak	−0.47	[−0.65; −0.29]	47%	0.11	Fixed	30
Glucose iPeak	−0.52	[−0.80; −0.25]	63%	0.29	Random	23
Insulin Peak	−0.29	[−0.44; −0.15]	0%	0	Fixed	23
Insulin iPeak	−0.24	[−0.41; −0.06]	0%	0	Fixed	18

## Data Availability

The data presented in this study will be made available on request from the corresponding author due to privacy.

## Supplementary Materials

The following supporting information can be downloaded at: https://www.mdpi.com/article/10.3390/nu17172840/s1, Table S1: Search terms and filters applied across different databases; Table S2: Study characteristics of clinical studies with postprandial glycemic response; Table S3: Study characteristics of clinical studies with chronic glycemic control; Table S4: Study characteristics of preclinical studies with reported chronic glycemic control; Table S5: The effect of AX consumption on chronic glycemic control from different source; Table S6: The effect of AX consumption on cardiometabolic indicators; Table S7: Sensitivity test of postprandial glucose AUC in clinical trials; Table S8: Sensitivity test of postprandial glucose iAUC in clinical studies; Table S9: Sensitivity test of postprandial insulin AUC in clinical studies; Table S10: Sensitivity test of postprandial insulin iAUC in clinical studies; Table S11: Sensitivity test of postprandial glucose Peak in clinical studies; Table S12: Sensitivity test of postprandial glucose iPeak in clinical studies; Table S13: Sensitivity test of postprandial insulin Peak in clinical studies; Table S14: Sensitivity test of postprandial insulin iPeak in clinical studies; Table S15: Sensitivity test of fasting glucose in preclinical studies; Table S16: Sensitivity test of fasting insulin in preclinical studies; Table S17: Sensitivity Test of HbA1c in preclinical studies; Table S18: Sensitivity test of HOMA-IR in preclinical studies; Table S19: Sensitivity test of fasting glucose in clinical studies; Table S20: Sensitivity test of fasting insulin in clinical studies; Table S21: Sensitivity test of HbA1c in clinical studies; Table S22: Sensitivity test of HOMA-IR in clinical studies; Figure S1: Risk of bias assessment of preclinical studies; Figure S2: Risk of bias assessment of clinical studies; Figure S3. Funnel plot of (A) Postprandial glucose AUC (Egger’s test *p* = 0.0038); (B) Postprandial glucose iAUC (Egger’s test *p* = 0.2091); (C) Postprandial insulin AUC (Egger’s test *p* = 0.4348); (D) Postprandial insulin iAUC (Egger’s test *p* = 0.9361); (E) Postprandial glucose Peak (Egger’s test *p* < 0.0001); (F) Postprandial glucose iPeak (Egger’s test *p* < 0.0001); (G) Postprandial insulin Peak (Egger’s test *p* = 0.3545); (H) Postprandial insulin iPeak (Egger’s test *p* = 0.7940); Figure S4. Funnel plot of (A) Fasting glucose (Egger’s test *p* < 0.0001); (B) Fasting insulin (Egger’s test *p* < 0.0001); (C) HOMA-IR (Egger’s test *p* < 0.0001) in preclinical studies; Figure S5: Funnel plot of (A) Fasting glucose (Egger’s test *p* < 0.0001); (B) Fasting insulin (Egger’s test *p* < 0.0001) in clinical studies.
