# Supplementary material for: Impact of Arabinoxylan Consumption on Glycemic Control: A Systematic Review and Meta-Analysis of Preclinical and Clinical Studies
Source: Nutrients. 2025 Aug 31;17(17):2840. doi: 10.3390/nu17172840 (PMC12429902; doi:10.3390/nu17172840)
Supplement: Supplementary file 1 [file nutrients-17-02840-s001.zip › nutrients-3844456-supplementary.pdf]

Table S1. Search terms and filters applied across different databases

Database	Search terms	Filter
Pubmed	(“Arabinoxylan”[Title/Abstract] OR “Wheat”[Title/Abstract] OR “Barley”[Title/Abstract] OR “Brewers’ spent grain”[Title/Abstract] OR “Corn”[Title/Abstract] OR “Maize”[Title/Abstract] OR “Rice”[Title/Abstract] OR “Rye”[Title/Abstract] OR “Oat”[Title/Abstract] OR “Sorghum”[Title/Abstract] OR “Millet”[Title/Abstract]) AND (“Glycemic control” [MeSH] OR “Glucose” [MeSH] OR “Insulin” [MeSH] OR “insulin resistance” [MeSH] OR “Glycated Hemoglobin”[MeSH] OR “Glycemic control” [Title/Abstract] OR “Glucose” [Title/Abstract] OR “Insulin” [Title/Abstract] OR “insulin resistance” [Title/Abstract] OR “Glycated Hemoglobin”[Title/Abstract] OR glycemic [Title/Abstract] OR “glucose response”[Title/Abstract] OR “postprandial glucose” [Title/Abstract] OR “postprandial insulin” [Title/Abstract] OR “insulin sensitivity” [Title/Abstract] OR “HOMA-IR” [Title/Abstract] OR “β-cell function” [Title/Abstract] OR “glucose-stimulated insulin secretion” [Title/Abstract] OR “Stumvoll index ” [Title/Abstract])	English, RCT, Humans, Other animals,
Embase	('arabinoxylan':ti,ab,kw OR 'wheat':ti,ab,kw OR 'barley':ti,ab,kw OR 'brewers spent grain':ti,ab,kw OR 'corn':ti,ab,kw OR 'maize':ti,ab,kw OR 'rice':ti,ab,kw OR 'rye':ti,ab,kw OR 'oat':ti,ab,kw OR 'sorghum':ti,ab,kw OR 'millet':ti,ab,kw) AND ('glycemic control':ti,ab,kw OR 'glycemic':ti,ab,kw OR 'glucose':ti,ab,kw OR 'glucose response':ti,ab,kw OR 'insulin':ti,ab,kw OR 'glycated hemoglobin':ti,ab,kw OR 'postprandial glucose':ti,ab,kw OR 'postprandial insulin':ti,ab,kw OR 'insulin sensitivity':ti,ab,kw OR 'insulin resistance':ti,ab,kw OR 'homa-ir':ti,ab,kw OR 'β-cell function':ti,ab,kw OR 'glucose-stimulated insulin secretion':ti,ab,kw OR 'stumvoll index':ti,ab,kw)	Embase (Not Medline) Human Nonhuman Article
COCHRANE	(“Arabinoxylan” OR “Wheat” OR “Barley” OR “Brewers’ spent grain” OR “Corn” OR “Maize” OR “Rice” OR “Rye” OR “Oat” OR “Sorghum” OR “Millet”):ti,ab,kw) AND ((MeSH “glycemic control” OR MeSH “Glucose” OR MeSH “Insulin” OR MeSH “Insulin resistance” OR MeSH “Glycated hemoglobin OR (“Glycemic control” OR “Glycemic” OR “Glucose” OR “Glucose response” OR “Insulin” OR “Glycated Hemoglobin” OR “Postprandial glucose” OR “postprandial insulin” OR “insulin sensitivity” OR “insulin resistance” OR “HOMA-IR” OR “β-cell function” OR “glucose-stimulated insulin secretion” OR “Stumvoll index”):ti,ab,kw))	In trials
CINAHL	(AB arabinoxylan OR AB wheat OR AB barley OR AB brewers spent grain OR AB corn OR AB maize OR AB rice OR AB rye OR AB oat OR AB sorghum OR AB millet) AND (MH Glycemic control OR MH Glucose OR MH Insulin OR	English Exclude Medline

	MH insulin sensitivity OR MH Glycated Hemoglobin OR Glycemic control OR Glycemic OR Glucose OR Glucose response OR Insulin OR Glycated Hemoglobin OR Postprandial glucose OR postprandial insulin OR insulin sensitivity OR insulin resistance OR HOMA-IR OR β-cell function OR glucose-stimulated insulin secretion OR Stumvoll index)	

Table S2. Study characteristics of clinical studies with postprandial glycemic response

Reference	Study design	Duration	Subject number (M/F)	Intervention food description	Control food description	Health status	Main Result
Hamberg et al. (1989)	Crossover	2 hours	8 (5/3)	36 g Wheat bran-enriched meal	Control meal	Healthy	Glucose iAUC<br>Insulin iAUC<br>Glucose Peak<br>Glucose iPeak<br>Insulin Peak<br>Insulin iPeak
Cherbut et al. (1994)	Crossover	2 hours	6 (6/0)	15 g wheat bran + 125 mL glucose drink	125 mL glucose drink	Healthy	Glucose iPeak<br>Insulin iPeak
Lia et al. (1997)	Crossover	7 hours	6 (3/3)	66g Oat bran-substituted meal	Control meal	Ileostomy subjects	Insulin iPeak
Lu et al. (2000)	Crossover	2 hours	14 (5/9)	6 g AX-substituted meal	Control meal	Healthy	Glucose iAUC<br>Insulin iAUC<br>Glucose Peak<br>Insulin Peak
Lu et al. (2000)	Crossover	2 hours	14 (5/9)	12 g AX-substituted meal	Control meal	Healthy	Glucose iAUC<br>Insulin iAUC<br>Glucose Peak<br>Insulin Peak
Juntunen et al. (2003)	Crossover	3 hours	19 (0/19)	Endosperm rye bread	Refined wheat bread	Healthy	Glucose AUC<br>Insulin AUC<br>Glucose iPeak<br>Insulin iPeak
Juntunen et al. (2003)	Crossover	3 hours	19 (0/19)	Traditional rye bread	Refined wheat bread	Healthy	Glucose AUC<br>Insulin AUC<br>Glucose iPeak<br>Insulin iPeak

Juntunen et al. (2003)	Crossover	3 hours	19 (0/19)	High-fiber rye bread	Refined wheat bread	Healthy	Glucose AUC<br>Insulin AUC<br>Glucose iPeak<br>Insulin iPeak
Möhlig et al. (2005)	Crossover	2 hours	11 (4/7)	6 g AX concentrate-substituted meal	Control meal	Healthy	Glucose iAUC<br>Insulin iAUC<br>Glucose Peak<br>Insulin Peak
Tapola et al. (2005)	Crossover	2 hours	12 (7/5)	62g Oat bran flour	12.5 g glucose drink	T2DM	Glucose Peak<br>Glucose iPeak
Tapola et al. (2005)	Crossover	2 hours	12 (7/5)	29g Oat bran crisp	12.5 g glucose drink	T2DM	Glucose Peak<br>Glucose iPeak
Tapola et al. (2005)	Crossover	2 hours	12 (7/5)	30g Oat bran flour + 25g glucose drink	25 g glucose drink	T2DM	Glucose Peak<br>Glucose iPeak
Hlebowicz et al., (2009)	Crossover	1.5 hours	10 (3/7)	Whole-meal rye bread	White bread	Healthy	Glucose AUC
Ulmius et al. (2009)	Crossover	2 hours	13 (6/7)	31 g Rye bran-substituted meal	Control meal	Healthy	Glucose iAUC<br>Insulin iAUC<br>Glucose Peak<br>Glucose iPeak<br>Insulin Peak<br>Insulin iPeak
Lappi et al. (2010)	Crossover	4 hours	13 (7/4)	Wholemeal wheat bread	White wheat bread	Insulin resistant subjects	Glucose AUC<br>Insulin AUC<br>Glucose iPeak<br>Insulin iPeak
Lappi et al. (2010)	Crossover	4 hours	13 (7/4)	Xylanase treated wholemeal wheat bread	White wheat bread	Insulin resistant subjects	Glucose AUC<br>Insulin AUC<br>Glucose iPeak<br>Insulin iPeak

Lappi et al. (2010)	Crossover	4 hours	13 (7/4)	Xylanase treated wholemeal wheat sourdough	White wheat bread	Insulin resistant subjects	Glucose AUC<br>Insulin AUC<br>Glucose iPeak<br>Insulin iPeak
Afagh et al. (2011)	Crossover	2 hours	9 (1/8)	Wheat bran-supplemented meal	Control meal	Imapired fasting glucose	Glucose AUC<br>Glucose Peak
Juvonen et al. (2011)	Crossover	3 hours	20 (5/15)	Wheat bran-substituted meal	Control meal	Healthy subject	Glucose Peak<br>Glucose iPeak<br>Insulin Peak<br>Insulin iPeak
Juvonen et al. (2011)	Crossover	3 hours	20 (5/15)	Oat bran-substituted meal	Control meal	Healthy subject	Glucose Peak<br>Glucose iPeak<br>Insulin Peak<br>Insulin iPeak
Juvonen et al. (2011)	Crossover	3 hours	20 (5/15)	Wheat and oat bran-substituted meal	Control meal	Healthy subject	Glucose Peak<br>Glucose iPeak<br>Insulin Peak<br>Insulin iPeak
Brennan et al. (2012)	Crossover	2 hours	12 (4/8)	15% oat bran substituted meal	Control meal	Healthy	Glucose iAUC<br>Glucose iPeak
Brennan et al. (2012)	Crossover	2 hours	12 (4/8)	15% psyllium substituted meal	Control meal	Healthy	Glucose iAUC<br>Glucose iPeak
Lappi et al. (2013)	Crossover	4 hours	15 (6/9)	Rye bread	White bread	Healthy	Insulin iAUC<br>Glucose Peak<br>Insulin Peak
Lappi et al. (2013)	Crossover	4 hours	15 (6/9)	Native rye bran substituted bread	White bread	Healthy	Insulin iAUC<br>Glucose Peak<br>Insulin Peak
Lappi et al. (2013)	Crossover	4 hours	15 (6/9)	Bioprocessed rye bran substituted bread	White bread	Healthy	Insulin iAUC<br>Glucose Peak<br>Insulin Peak

Hartvigsen et al. (2014)a	Crossover	2 hours	15 (7/8)	AX-enriched bread	White bread	Metabolic syndrome	Glucose iAUC<br>Insulin iAUC<br>Glucose Peak<br>Insulin Peak
Hartvigsen et al. (2014)a	Crossover	2 hours	15 (7/8)	Rye bread	White bread	Metabolic syndrome	Glucose iAUC<br>Insulin iAUC<br>Glucose Peak<br>Insulin Peak
Hartvigsen et al. (2014)b	Crossover	2 hours	15 (7/8)	AX-enriched porridge	Control porridge	Metabolic syndrome	Glucose iAUC<br>Insulin iAUC<br>Glucose Peak<br>Insulin Peak
Hartvigsen et al. (2014)b	Crossover	2 hours	15 (7/8)	AX and rye kernels enriched porridge	Control porridge	Metabolic syndrome	Glucose iAUC<br>Insulin iAUC<br>Glucose Peak<br>Insulin Peak
Hartvigsen et al. (2014)b	Crossover	2 hours	15 (7/8)	Rye kernels enriched porridge	Control porridge	Metabolic syndrome	Glucose iAUC<br>Insulin iAUC<br>Glucose Peak<br>Insulin Peak
Lappi et al. (2014)	Crossover	2 hours	21 (9/12)	Rye bread	White bread	Mild gastrointestinal symptoms subjects	Glucose Peak<br>Insulin Peak
Lappi et al. (2014)	Crossover	2 hours	21 (9/12)	Bioprocessed rye bran substituted bread	White bread	Mild gastrointestinal symptoms subjects	Glucose Peak<br>Insulin Peak
Lafond et al. (2015)	Crossover	2 hours	23 (7/16)	15 g AXOS-enriched meal	White bread	Overweight women	Glucose iAUC<br>Insulin iAUC
Lafond et al. (2015)	Crossover	2 hours	23 (7/16)	15 g AX-enriched meal	White bread	Overweight women	Glucose iAUC<br>Insulin iAUC

Giulia et al. (2016)	Crossover	4 hours	24 (18/6)	AX-substituted bread	White bread	Healthy	Glucose Peak
Lee et al. (2016)	Crossover	2 hours	21 (11/10)	40 g rye flakes-substituted meal	Control meal	Healthy	Glucose AUC
Lee et al. (2016)	Crossover	2 hours	21 (11/10)	55 g rye flakes-substituted meal	Control meal	Healthy	Glucose AUC
Shi et al. (2017)	Crossover	1.5 hours	21 (11/10)	40 g whole-grain rye	Control meal	Healthy	Glucose AUC
Shi et al. (2017)	Crossover	1.5 hours	21 (11/10)	55 g whole-grain rye	Control meal	Healthy	Glucose AUC
Camps et al. (2018)	Crossover	3 hours	15 (15/0)	Rice bran-enriched soymilk	Control meal	Healthy	Glucose iAUC<br>Insulin iAUC<br>Glucose iPeak<br>Insulin iPeak
Ullah et al. (2022)	Crossover	2 hours	15 (15/0)	BSG-substituted meal	Control meal	Impaired glucose tolerance	Glucose Peak<br>Glucose iPeak<br>Insulin Peak<br>Insulin iPeak
Ullah et al. (2022)	Crossover	2 hours	25 (0/25)	BSG-substituted meal	Control meal	Impaired glucose tolerance	Glucose Peak<br>Glucose iPeak<br>Insulin Peak<br>Insulin iPeak
Åberg et al. (2024)	Crossover	4 hours	21	Rye-substituted meal	Control meal	Obesity	Glucose iAUC<br>Glucose Peak
Ponzo et al. (2024)	Crossover	2 hours	10 (6/4)	BSG-substituted meal	Control meal	Healthy	Glucose AUC<br>Insulin AUC<br>Glucose Peak<br>Insulin Peak
Moreira (2024)	Crossover	2 hours	19 (19/0)	15g wheat bran-substituted meal	Control meal	T2DM	Glucose AUC<br>Glucose Peak<br>Glucose iPeak

Xu et al. (2024)	Crossover	4 hours	15 (10/5)	BSG-substituted biscuits	Control biscuits	Metabolic syndrome	Glucose AUC<br>Glucose iAUC<br>Insulin AUC<br>Insulin iAUC<br>Glucose Peak<br>Glucose iPeak<br>Insulin Peak<br>Insulin iPeak
Xu et al. (2024)	Crossover	4 hours	15 (10/5)	Fermented BSG-substituted biscuits	Control biscuits	Metabolic syndrome	Glucose AUC<br>Glucose iAUC<br>Insulin AUC<br>Insulin iAUC<br>Glucose Peak<br>Glucose iPeak<br>Insulin Peak<br>Insulin iPeak

Table S3. Study characteristics of clinical studies with chronic glycemic control

Reference	Study design	Duration	Subject number (Male/Female)	Intervention food description	Control food description	Health status	Main Result
Anderson et al. (1984)	Parallel	3 weeks	20 (20/0)<br>I: n = 10<br>C: n = 10	100g Oat bran-substituted diet	Bean diet	Hypercholesterole mic	Fasting glucose
Vaaler et al. (1986)	Crossover	12 weeks	28(15/13)	33g Wheat bran-substituted diet	Guar gum-enriched diet	Insulin-dependent diabetics	Fasting glucose<br>HbA1c
Jenkins et al. (2002)	Crossover	12 weeks	23 (16/7)	19g Wheat bran-substituted diet	Normal diet	Type 2 diabetic mellites	Fasting glucose<br>HbA1c
Juntunen et al. (2003)	Crossover	8 weeks	20 (0/20)	Rye bread	White bread	healthy postmenopausal women	Fasting glucose<br>Fasting insulin
McIntosh et al. (2003)	Crossover	4 weeks	28 (28/0)	Wheat fiber-substituted diet	Low fiber diet	Overweight	Fasting glucose
McIntosh et al. (2003)	Crossover	4 weeks	28 (28/0)	Rye fiber-substituted diet	Low fiber diet	Overweight	Fasting glucose
Lu et al. (2004)	Crossover	5 weeks	15 (9/6)	14% AX-substituted diet	Control diet	Type 2 diabetic mellites	Fasting glucose<br>Fasting insulin
Garcia et al. (2007)	Crossover	6 weeks	11 (4/7)	15g AX concentrate supplemented	15g Placebo	impaired glucose tolerance	Fasting glucose<br>Fasting insulin
Ulmius et al. (2009)	Crossover	1 week	13 (6/7)	Rye bran-substituted diet	Control diet	Healthy subject	Fasting glucose<br>Fasting insulin
Cheng et al. (2010)	Parallel	12 weeks	28 (13/15)<br>I: n = 17<br>C: n = 11	Rice bran flour-substituted diet	Rice flour diet	Type 2 diabetic mellites	Fasting glucose<br>Fasting insulin<br>HbA1c<br>HOMA-IR

Maki et al. (2012)	Crossover	3 weeks	65 (30/35)	2.2g AXOS-supplemented diet	Control diet	Healthy subject	Fasting glucose<br>Fasting insulin
Maki et al. (2012)	Crossover	3 weeks	65 (30/35)	4.4g AXOS-supplemented diet	Control diet	Healthy subject	Fasting glucose<br>Fasting insulin
Raimondi et al. (2016)	Parallel	12 weeks	132 (44/88)<br>I: n = 66<br>C: n = 66	40 g Oat bran supplemented	40 g Placebo	hypercholesterolemic subject	Fasting glucose<br>Fasting insulin<br>HOMA-IR
Aoe et al. (2018)	Parallel	4 weeks	30 (20/10)<br>I: n = 15<br>C: n = 15	Wheat bran-substituted cereal bar	Control cereal bar	Healthy subject	Fasting glucose<br>HbA1c
Salden et al. (2018)	Parallel	6 weeks	30 (18/12)<br>I: n = 16<br>C: n = 14	7.5 g AX supplemented	15 g Placebo	Overweight and obese	Fasting glucose<br>Fasting insulin
Salden et al. (2018)	Parallel	6 weeks	31 (15/16)<br>I: n = 17<br>C: n = 14	15 g AX supplemented	15 g Placebo	Overweight and obese	Fasting glucose<br>Fasting insulin
Schioldan et al. (2018)	Crossover	4 weeks	19 (14/5)	Wheat bran-substituted diet	Western-style diet	Metabolic syndrome	Fasting glucose<br>Fasting insulin<br>HOMA-IR
Müller et al. (2020)	Parallel	12 weeks	48 (12/36)<br>I: n = 24<br>C: n = 24	15g AXOS concentrate supplemented	15g placebo	Healthy	Fasting glucose<br>Fasting insulin
Xue et al. (2020)	Parallel	12 weeks	158 (32/126)<br>I: n = 80<br>C: n = 78	Fermented rye bran-substituted diet	Control diet	Healthy	Fasting glucose<br>Fasting insulin<br>HbA1c<br>HOMA-IR

Liu et al. (2021)	Parallel	12 weeks	84 (17/67)<br>I: n = 31<br>C: n = 53	Fermented rye bran-substituted diet	Control diet	Healthy	Fasting glucose<br>Fasting insulin<br>HbA1c
Saphyakhajorn et al. (2022)	Parallel	12 weeks	61 (17/44)<br>I: n = 30<br>C: n = 31	30 g defatted rice bran supplemented	30 g placebo	Overweight and hypercholesterolemic	Fasting glucose<br>Fasting insulin<br>HbA1c<br>HOMA-IR
Schmidt-Combest et al. (2023)	Parallel	8 weeks	37 (9/28)<br>I: n = 19<br>C: n = 18	8.3 g BSG-substituted muffin	Control muffin	Healthy subject	Fasting glucose<br>Fasting insulin
Ghorbani et al. (2024)	Parallel	8 weeks	43 (21/22)<br>I: n = 24<br>C: n = 19	15g Black rice bran powder-substituted diet	Control diet	Metabolic syndrome	Fasting glucose

Table S4. Study characteristics of preclinical studies with reported chronic glycemic control

Reference	Study design	Duration	Animal type	Sample size (Male/Female)	Intervention food description	Control food description	Health status	Main Result	Underlying mechanisms
Ohara et al. (2000)	Parallel	8 weeks	Rat	11 (11/0)<br>I: n = 5<br>C: n = 6	0.5g/kg bw modified rice bran + control diet	Control diet	Healthy	Fasting glucose<br>Fasting insulin	N.A.
Ohara et al. (2000)	Parallel	8 weeks	Rat	11 (11/0)<br>I: n = 6<br>C: n = 5	0.5g/kg bw modified rice bran + control diet	Control diet	Streptozotocin-induced diabetes	Fasting glucose<br>Fasting insulin	N.A.
Lærke et al. (2008)	Parallel	1.7 weeks	Pig	17 (0/17)<br>I: n = 9<br>C: n = 8	Rye bun diet	Wheat bun diet	Hypercholesterolaemic	Fasting glucose<br>Fasting insulin	N.A.
Kim et al. (2010)	Parallel	7 weeks	Mice	16 (16/0)<br>I: n = 8<br>C: n = 8	30% E rice bran-substituted high-fat diet	High-fat diet	Healthy	Fasting glucose<br>Fasting insulin	Activity of hepatic enzymes:<br>Glucokinase ↑<br>Phosphoenolpyruvate carboxykinase ↓<br>Glucose-6-phosphatase ↓
Neyrinck et al. (2012)	Parallel	8 weeks	Mice	16 (16/0)<br>I: n = 8<br>C: n = 8	7.5% AXOS-supplemented diet	Control diet	C57bl6/J mice	Fasting glucose<br>Fasting insulin<br>HOMA-IR	N.A.
Hartvigsen et al. (2013)	Parallel	6 weeks	Rat	24 (24/0)<br>I: n = 12<br>C: n = 12	AX-substituted bread	White bread	ZDF rat (Obese)	Fasting glucose<br>Fasting insulin<br>HbA1c	Expression of genes:<br>Adipose glyceraldehyde-3-phosphate dehydrogenase ↑<br>Adipose AMP-activated protein kinase ↑

Hartvigsen et al. (2013)	Parallel	6 weeks	Rat	24 (24/0)<br>I: n = 12<br>C: n = 12	Rye-substituted bread	White bread	ZDF rat (Obese)	Fasting glucose<br>Fasting insulin<br>HbA1c	Expression of genes:<br>Adipose glyceraldehyde-3-phosphate dehydrogenase ↑<br>Adipose adiponectin receptors-1 ↑<br>Adipose AMP-activated protein kinase ↑
Hartvigsen et al. (2013)	Parallel	6 weeks	Rat	24 (24/0)<br>I: n = 12<br>C: n = 12	Whole-meal bread	White bread	ZDF rat (Obese)	Fasting glucose<br>Fasting insulin<br>HbA1c	Expression of genes:<br>Adipose glyceraldehyde-3-phosphate dehydrogenase ↑<br>Adipose AMP-activated protein kinase ↑
Abulnaja et al. (2015)	Parallel	8 weeks	Rat	12 (12/0)<br>I: n = 6<br>C: n = 6	5% barley bran-substituted diet	Control diet	Hypercholesterolemia	Fasting glucose<br>HbA1c	N. A.
Abulnaja et al. (2015)	Parallel	8 weeks	Rat	12 (12/0)<br>I: n = 6<br>C: n = 6	10% barley bran-substituted diet	Control diet	Hypercholesterolemia	Fasting glucose<br>HbA1c	N. A.
Agyekum et al. (2015)	Parallel	3 weeks	Pig	16 (8/8)<br>I: n = 8<br>C: n = 8	6.6% E dried grain diet	Control diet	Healthy	Fasting glucose	Expression of genes:<br>Ileal monocarboxylate transporter 1 ↑
Nielsen et al. (2015)	Parallel	3 weeks	Pig	20 (0/20)<br>I: n = 10<br>C: n = 10	72g/kg bw arabinoxylan-substituted western-style diet	Western-style diet	Healthy	Fasting insulin<br>HOMA-IR	Concentrations of plasma short chain fatty acids ↑<br>Adipose monocarboxylate

								transporter 1 expression – Adipose AMP-activated protein kinase expression –
Alauddin et al. (2016)	Parallel	4 weeks	Rat	12 (12/0) I: n = 6 C: n = 6	5% rice bran-substituted diet	Control diet	SHRSP/Izm rats (hypertension)	Fasting glucose Fasting insulin HOMA-IR	Expression of genes: Hepatic phosphoenolpyruvate carboxykinase ↓
Alauddin et al. (2016)	Parallel	4 weeks	Rat	12 (12/0) I: n = 6 C: n = 6	5% fermented rice bran-substituted diet	Control diet	SHRSP/Izm rats (hypertension)	Fasting glucose Fasting insulin HOMA-IR	Expression of genes: Hepatic glucose-6-phosphatase ↓ Hepatic phosphoenolpyruvate carboxykinase ↓ Hepatic AMP-activated protein kinase ↑
Kieffer et al. (2016)	Parallel	10 weeks	Mice	30(30/0) I: n = 15 C: n = 15	20% E enzyme-treated wheat bran-substituted diet	Control diet	C57BL/6J mice (Healthy)	Fasting glucose Fasting insulin	Expression of genes: Protein phosphatase 1, regulatory subunit 3B & 3C ↑
Zhang et al. (2016)	Parallel	24 weeks	Mice	24(24/0) I: n = 12 C: n = 12	High fat diet with 0.8% wheat bran fiber	High-fat diet	C57BL/6J mice (Healthy)	Fasting insulin	Expression of genes: Adipose leptin receptor ↑
Neyrinck et al. (2018)	Parallel	4 weeks	Mice	18 (18/0) I: n = 9 C: n = 9	5% AXOS + high-fat diet	High-fat diet	C57BL6 mice (Healthy)	Fasting glucose Fasting insulin HOMA-IR	The abundance of Bifidobacteria ↑
Nie et al. (2018)	Parallel	4 weeks	Rat	12 (12/0) I: n = 6 C: n = 6	200 mg/kg bw AX + Control diet	Control diet	Healthy	Fasting glucose Fasting insulin	Untargeted metabolomics analysis

									Alterations in gut microbiota composition
Nie et al. (2018)	Parallel	4 weeks	Rat	12 (12/0)<br>I: n = 6<br>C: n = 6	100 mg/kg bw AX + Control diet	Control diet	T2DM	Fasting glucose<br>Fasting insulin	Untargeted metabolomics analysis<br>Alterations in gut microbiota composition
Nie et al. (2018)	Parallel	4 weeks	Rat	12 (12/0)<br>I: n = 6<br>C: n = 6	200 mg/kg bw AX + Control diet	Control diet	T2DM	Fasting glucose<br>Fasting insulin	Untargeted metabolomics analysis<br>Alterations in gut microbiota composition
Nie et al. (2018)	Parallel	4 weeks	Rat	12 (12/0)<br>I: n = 6<br>C: n = 6	400 mg/kg bw AX + Control diet	Control diet	T2DM	Fasting glucose<br>Fasting insulin	Untargeted metabolomics analysis<br>Alterations in gut microbiota composition
Sarma et al. (2018)	Parallel	10 weeks	Mice	12 (12/0)<br>I: n = 6<br>C: n = 6	1g/kg bw AX + normal diet	Normal diet	Swiss albino mice (Healthy)	Fasting glucose<br>Fasting insulin	Alterations in gut microbiota composition<br>Concentration of cecal propionate↑
Sarma et al. (2018)	Parallel	10 weeks	Mice	12 (12/0)<br>I: n = 6<br>C: n = 6	0.5g/kg bw AX + high-fat diet	high-fat diet	Swiss albino mice (Healthy)	Fasting glucose<br>Fasting insulin	Alterations in gut microbiota composition<br>Concentration of cecal propionate↑

Sarma et al. (2018)	Parallel	10 weeks	Mice	12 (12/0)<br>I: n = 6<br>C: n = 6	1g/kg bw AX + high-fat diet	high-fat diet	Swiss albino mice (Healthy)	Fasting glucose<br>Fasting insulin	Alterations in gut microbiota composition<br>Concentration of cecal propionate↑
Li et al. (2019)	Parallel	4 weeks	Rat	12<br>I: n = 6<br>C: n = 6	270 mg/kg diet + normal diet	Normal diet	Wistar rats (Healthy)	Fasting glucose<br>Fasting insulin<br>HOMA-IR	Concentration of cecal short chain fatty acids↑
Li et al. (2019)	Parallel	4 weeks	Rat	12<br>I: n = 6<br>C: n = 6	270 mg/kg diet + high-fat diet	High-fat diet	Wistar rats (T2DM)	Fasting glucose<br>Fasting insulin<br>HOMA-IR	Concentration of cecal short chain fatty acids↑
Yang et al. (2019)	Parallel	8 weeks	Rat	20 (0/20)<br>I: n = 10<br>C: n = 10	2% E rice bran + high energy diet	High energy diet	Sprague–Dawley rats (Healthy)	Fasting glucose	N. A.
Yang et al. (2019)	Parallel	8 weeks	Rat	20 (0/20)<br>I: n = 10<br>C: n = 10	4% E rice bran + high energy diet	High energy diet	Sprague–Dawley rats (Healthy)	Fasting glucose	N. A.
Yang et al. (2019)	Parallel	8 weeks	Rat	20 (0/20)<br>I: n = 10<br>C: n = 10	8% E rice bran + high energy diet	High energy diet	Sprague–Dawley rats (Healthy)	Fasting glucose	N. A.
Chen et al. (2020)	Parallel	8 weeks	Mice	20 (20/0)<br>I: n = 10<br>C: n = 10	4% AX + high-fat diet	High-fat diet	ICR/KM mice (Healthy)	Fasting glucose	Alterations in gut microbiota composition
Ai et al. (2021)	Parallel	8 weeks	Rat	20 (20/0)<br>I: n = 10<br>C: n = 10	1.0 g/kg bw rice bran + high-fat diet	High-fat diet	Streptozotocin-induced diabetes	Fasting glucose	Alterations in gut microbiota composition
Ai et al. (2021)	Parallel	8 weeks	Rat	20 (20/0)<br>I: n = 10<br>C: n = 10	0.5g/kg bw fermented rice bran + high-fat diet	High-fat diet	Streptozotocin-induced diabetes	Fasting glucose	Alterations in gut microbiota composition
Ai et al. (2021)	Parallel	8 weeks	Rat	20 (20/0)<br>I: n = 10<br>C: n = 10	1.0 g/kg bw fermented rice bran + high-fat diet	High-fat diet	Streptozotocin-induced diabetes	Fasting glucose	Alterations in gut microbiota composition

Nie et al. (2021)	Parallel	8 weeks	Rat	30 (30/0)<br>I: n = 15<br>C: n = 15	270 mg/kg bw AX + control diet	Control diet	Wistar rats (T2DM)	Fasting glucose<br>Fasting insulin<br>HOMA-IR	Alterations in gut microbiota composition
Alzahrani et al. (2022)	Parallel	8 weeks	Mice	12 (12/0)<br>I: n = 6<br>C: n = 6	25 mg/kg bw whole pearl millet grain powder ethanol extract + high-fat diet	High-fat diet	Obesity, IR, and NAFLD mice	Fasting glucose<br>Fasting insulin<br>HOMA-IR	N. A.
Alzahrani et al. (2022)	Parallel	8 weeks	Mice	12 (12/0)<br>I: n = 6<br>C: n = 6	50 mg/kg bw whole pearl millet grain powder ethanol extract + high-fat diet	High-fat diet	Obesity, IR, and NAFLD mice	Fasting glucose<br>Fasting insulin<br>HOMA-IR	N. A.
Alzahrani et al. (2022)	Parallel	8 weeks	Mice	12 (12/0)<br>I: n = 6<br>C: n = 6	100 mg/kg bw whole pearl millet grain powder ethanol extract + high-fat diet	High-fat diet	Obesity, IR, and NAFLD mice	Fasting glucose<br>Fasting insulin<br>HOMA-IR	N. A.
Alzahrani et al. (2022)	Parallel	8 weeks	Mice	12 (12/0)<br>I: n = 6<br>C: n = 6	10% E whole pearl millet grain powder+ high-fat diet	High-fat diet	Obesity, IR, and NAFLD mice	Fasting glucose<br>Fasting insulin<br>HOMA-IR	N. A.
Alzahrani et al. (2022)	Parallel	8 weeks	Mice	12 (12/0)<br>I: n = 6<br>C: n = 6	20% E whole pearl millet grain powder+ high-fat diet	High-fat diet	Obesity, IR, and NAFLD mice	Fasting glucose<br>Fasting insulin<br>HOMA-IR	N. A.
Alzahrani et al. (2022)	Parallel	8 weeks	Mice	12 (12/0)<br>I: n = 6<br>C: n = 6	30% E whole pearl millet grain powder+ high-fat diet	High-fat diet	Obesity, IR, and NAFLD mice	Fasting glucose<br>Fasting insulin<br>HOMA-IR	N. A.

An et al. (2022)	Parallel	6 weeks	Broiler	84 (84/0)<br>I: n = 42<br>C: n = 42	7% dry fermented wheat bran + sterile saline	Basel diet + sterile saline	Healthy	Fasting glucose	Alterations in gut microbiota composition
An et al. (2022)	Parallel	6 weeks	Broiler	84 (84/0)<br>I: n = 42<br>C: n = 42	7% wet fermented wheat bran + sterile saline	Basel diet + sterile saline	Healthy	Fasting glucose	Alterations in gut microbiota composition
An et al. (2022)	Parallel	6 weeks	Broiler	84 (84/0)<br>I: n = 42<br>C: n = 42	7% dry fermented wheat bran + lipopolysaccharide	Basel diet + lipopolysacch aride	Healthy	Fasting glucose	Alterations in gut microbiota composition
An et al. (2022)	Parallel	6 weeks	Broiler	84 (84/0)<br>I: n = 42<br>C: n = 42	7% wet fermented wheat bran + lipopolysaccharide	Basel diet + lipopolysacch aride	Healthy	Fasting glucose	Alterations in gut microbiota composition
Mio et al. (2022)	Parallel	12 weeks	Mice	16 (16/0)<br>I: n = 8<br>C: n = 8	271.2 g/kg diet AX-containing barley flour + control diet	Control diet	C57BL/6J mice (Healthy)	HOMA-IR	Concentration of short chain fatty acids in cecal ↑
Abdou et al. (2023)	Parallel	4 weeks	Rat	12 (12/0)<br>I: n = 6<br>C: n = 6	100 mg/kg bw AX + control diet	Control diet	Healthy	Fasting glucose<br>Fasting insulin	N. A.
Abdou et al. (2023)	Parallel	4 weeks	Rat	12 (12/0)<br>I: n = 6<br>C: n = 6	100 mg/kg bw AX + high-fat diet	High-fat diet	Streptozoto cin-induced diabetes	Fasting glucose<br>Fasting insulin	N. A.
Fang et al. (2024)	Parallel	18 weeks	Mice	24 (24/0)<br>I: n = 12<br>C: n = 12	5% AX + high-fat diet	High-fat diet	C57BL/6 J mice (Healthy)	Fasting glucose	Alterations in gut microbiota composition
Agista et al. (2024)	Parallel	4 weeks	Mice	15 (15/0)<br>I: n = 7<br>C: n = 8	10% rice bran + control diet	Control diet	KK-Ay/TaJcl mice (Obese)	Fasting glucose<br>Fasting insulin<br>HOMA-IR	N. A.
Agista et al. (2024)	Parallel	4 weeks	Mice	17 (17/0)<br>I: n = 9<br>C: n = 8	10% fermented rice bran + control diet	Control diet	KK-Ay/TaJcl	Fasting glucose<br>Fasting insulin<br>HOMA-IR	N. A.

mice<br>(Obese)

Table S5. The effect of AX consumption on chronic glycemic control from different source

**Indicators**	**SMD**	**95% CI**	**I^2**	**τ^2**	**Model**	**Comparisons included (*n*)**
Preclinical studies						
Fasting glucose						
Overall	-1.18	[-1.56, -0.49]	82	1.36	Random	46
Extracted AX	-1.09	[-1.70, -0.49]				27
Intrinsic AX	-1.24	[-1.74, -0.74]				19
Fasting insulin						
Overall	-1.07	[-1.92, -0.23]	89	5.46	Random	32
Extracted AX	-1.14	[-2.25, -0.02]				18
Intrinsic AX	-1.01	[-2.36, 0.35]				14
HbA1c						
Overall	-2.93	[-5.48, -0.38]	94	7.92	Random	5
Extracted AX	-5.37	[-7.07, -3.67]				1
Intrinsic AX	-2.35	[-5.24, 0.55]				4
HOMA-IR						
Overall	-2.44	[-3.66, -1.22]	86	5.47	Random	16
Extracted AX	-2.35	[-4.25, -0.46]				7
Intrinsic AX	-2.55	[-4.28, -0.82]				9
Clinical studies						
Fasting glucose						
Overall	-0.10	[-0.16, -0.03]	61	0.01	Random	23
Extracted AX	-0.15	[-0.24, -0.06]				8
Intrinsic AX	-0.05	[-0.13, 0.03]				15
Fasting insulin						
Overall	0.02	[-5.89, 5.93]	67	84.16	Random	15
Extracted AX	-6.18	[-17.18, 4.83]				7
Intrinsic AX	3.47	[-2.79, 9.73]				8
HbA1c						
Overall	-0.01	[-0.03, 0.01]	39	0.00	Fixed	7
Extracted AX	-	-				
Intrinsic AX	-0.01	[-0.03, 0.01]				7
HOMA-IR						
Overall	-0.02	[-0.35, 0.31]	67	0.08	Random	5
Extracted AX	-0.30	[-1.03, 0.43]				1
Intrinsic AX	0.03	[-0.35, 0.41]				4

Table S6. The effect of AX consumption on cardiometabolic indicators

Outcomes	SMD	95% CI	I^2	τ^2	Model	Comparisons included (*n*)
			Preclinical studies			
TC						
Overall	-1.28	[-1.80, -0.76]	83.7	2.58	Random	43
Metabolically impaired	-1.69	[-2.46, -0.92]				24
Metabolically healthy	-0.50	[-0.91, -0.76]				19
LDL-C						
Overall	-1.70	[-2.47, -0.94]	84.3	3.82	Random	29
Metabolically impaired	-1.69	[-2.55, -0.83]				17
Metabolically healthy	-1.97	[-2.47, -0.94]				12
HDL-C						
Overall	0.80	[0.20, 1.41]	84.9%	2.88	Random	34
Metabolically impaired	1.21	[0.31, 2.11]				21
Metabolically healthy	0.06	[-0.28, 0.41]				13
TG						
Overall	-1.10	[-1.66, -0.53]	85	3.18	Random	45
Metabolically impaired	-1.42	[-2.41, -0.42]				24
Metabolically healthy	-0.68	[-1.21, -0.16]				21
			Clinical studies			
TC						
Overall	-0.12	[-0.43, 0.19]	92	0.35	Random	14
Metabolically impaired	-0.19	[-0.73, 0.36]				8
Metabolically healthy	-0.06	[-0.25, 0.12]				6
LDL-C						
Overall	-0.12	[-0.29, 0.05]	92.3	0.10	Random	14
Metabolically impaired	-0.16	[-0.44, 0.12]				8
Metabolically healthy	-0.08	[-0.26, 0.09]				6
HDL-C						
Overall	0.02	[0.00, 0.05]	60.6	0.00	Random	16
Metabolically impaired	0.02	[-0.01, 0.05]				11
Metabolically healthy	0.01	[-0.02, 0.04]				5
TG						
Overall	-0.02	[-0.11, 0.08]	42.5	0.01	Fixed	17
Metabolically impaired	-0.08	[-0.26, 0.11]				11
Metabolically healthy	0.02	[-0.08, 0.13]				6
BMI						
Overall	-0.08	[-0.32, 0.16]	71.4	0.04	Random	6
Metabolically impaired	0.11	[-0.05, 0.28]				3
Metabolically healthy	-0.18	[-0.51, 0.15]				3
SBP						
Overall	-0.92	[-3.93, 2.10]	72.3	11.6	Random	7
Metabolically impaired	-0.09	[-3.07, 2.89]				6
Metabolically healthy	-5.80	[-10.27, -1.33]				1
DBP						
Overall	-0.80	[-3.04, 1.45]	71.5	6.59	Random	7
Metabolically impaired	-0.44	[-2.88, 1.99]				6
Metabolically healthy	-3.40	[-7.41, 0.61]				1

Table S7. Sensitivity test of postprandial glucose AUC in clinical trials

Study omitted	SMD	95% CI	I^2 (%)
Overall	-0.34	[-0.58; -0.10]	51%
Juntunen (i) 2003	-0.37	[-0.62, -0.11]	52%
Juntunen (ii) 2003	-0.34	[-0.60, -0.09]	54%
Juntunen (iii) 2003	-0.36	[-0.61, -0.10]	54%
Tapola (i) 2005	-0.28	[-0.50, -0.05]	41%
Tapola (ii) 2005	-0.33	[-0.58, -0.08]	53%
Tapola (iii) 2005	-0.33	[-0.58, -0.08]	53%
Hlebowicz 2009	-0.36	[-0.61, -0.11]	53%
Lappi (i) 2010	-0.33	[-0.58, -0.08]	53%
Lappi (ii) 2010	-0.34	[-0.59, -0.09]	54%
Lappi (iii) 2010	-0.32	[-0.57, -0.07]	52%
Afagh 2011	-0.35	[-0.60, -0.10]	54%
Lee (i) 2016	-0.34	[-0.60, -0.09]	54%
Lee (ii) 2016	-0.39	[-0.63, -0.16]	43%
Shi (i) 2017	-0.37	[-0.63, -0.12]	52%
Shi (ii) 2017	-0.39	[-0.63, -0.16]	43%
Ponzo 2024	-0.32	[-0.57, -0.07]	52%
Xu (i) 2024	-0.35	[-0.61, -0.10]	54%
Xu (ii) 2024	-0.35	[-0.60, -0.09]	54%
Moreira 2024	-0.27	[-0.50, -0.05]	40%

Table S8. Sensitivity test of postprandial glucose iAUC in clinical studies

Study omitted	SMD	95% CI	I^2 (%)
Overall	-0.41	[-0.57; -0.25]	0%
Hamberg 1989	-0.40	[-0.57, -0.23]	0%
Lu (i) 2000	-0.41	[-0.57, -0.24]	0%
Lu (ii) 2000	-0.39	[-0.56, -0.22]	0%
Mohlig 2005	-0.41	[-0.58, -0.25]	0%
Ulmius 2009	-0.40	[-0.57, -0.24]	0%
Brennan (i) 2012	-0.45	[-0.62, -0.28]	0%
Brennan (ii) 2012	-0.39	[-0.56, -0.22]	0%
Hartvigsen (i) 2014a	-0.41	[-0.58, -0.24]	0%
Hartvigsen (ii) 2014a	-0.40	[-0.56, -0.23]	0%
Hartvigsen (i) 2014b	-0.41	[-0.57, -0.24]	0%
Hartvigsen (ii) 2014b	-0.40	[-0.57, -0.23]	0%
Hartvigsen (iii) 2014b	-0.41	[-0.58, -0.24]	0%
Lafond 2015	-0.44	[-0.62, -0.27]	0%
Lafond 2015	-0.43	[-0.61, -0.26]	0%
Stefan 2018	-0.40	[-0.56, -0.23]	0%
Xu (i) 2024	-0.41	[-0.58, -0.24]	0%
Xu (ii) 2024	-0.41	[-0.57, -0.24]	0%
Aberg 2024	-0.40	[-0.57, -0.23]	0%

Table S9. Sensitivity test of postprandial insulin AUC in clinical studies

Study omitted	SMD	95% CI	I^2 (%)
Overall	-0.42	[-0.67; -0.17]	0%
Juntunen (i) 2003	-0.36	[-0.63, -0.09]	0%
Juntunen (ii) 2003	-0.37	[-0.64, -0.11]	0%
Juntunen (iii) 2004	-0.41	[-0.68, -0.15]	0%
Lappi (i) 2010	-0.44	[-0.70, -0.19]	0%
Lappi (ii) 2010	-0.45	[-0.71, -0.19]	0%
Lappi (iii) 2010	-0.42	[-0.68, -0.16]	0%
Ponzo 2024	-0.40	[-0.65, -0.14]	0%
Xu (i) 2024	-0.47	[-0.73, -0.21]	0%
Xu (ii) 2024	-0.43	[-0.69, -0.16]	0%

Table S10. Sensitivity test of postprandial insulin iAUC in clinical studies

Study omitted	SMD	95% CI	I^2 (%)
Overall	-0.28	[-0.44; -0.12]	0%
Hamberg 1989	-0.28	[-0.44, -0.11]	0%
Lu (i) 2000	-0.28	[-0.45, -0.11]	0%
Lu (ii) 2000	-0.27	[-0.44, -0.1]	0%
Mohlig 2005	-0.30	[-0.47, -0.14]	0%
Ulmius 2009	-0.28	[-0.45, -0.12]	0%
Lappi (i) 2013	-0.26	[-0.42, -0.09]	0%
Lappi (ii) 2013	-0.29	[-0.46, -0.12]	0%
Lappi (iii) 2013	-0.28	[-0.45, -0.11]	0%
Hartvigsen (i) 2014a	-0.30	[-0.47, -0.14]	0%
Hartvigsen (ii) 2014a	-0.25	[-0.41, -0.08]	0%
Hartvigsen (i) 2014b	-0.26	[-0.43, -0.09]	0%
Hartvigsen (ii) 2014b	-0.28	[-0.44, -0.11]	0%
Hartvigsen (iii) 2014b	-0.27	[-0.43, -0.10]	0%
Lafond 2015	-0.28	[-0.46, -0.11]	0%
Lafond 2015	-0.27	[-0.45, -0.10]	0%
Stefan 2018	-0.30	[-0.47, -0.14]	0%
Xu (i) 2024	-0.29	[-0.46, -0.12]	0%
Xu (ii) 2024	-0.28	[-0.45, -0.11]	0%

Table S11. Sensitivity test of postprandial glucose Peak in clinical studies

Study omitted	SMD	95% CI	I^2 (%)
Overall	-0.47	[-0.65; -0.29]	47%
Hamberg 1989	-0.47	[-0.65, -0.29]	47%
Lu (i) 2000	-0.46	[-0.65, -0.28]	46%
Lu (ii) 2000	-0.44	[-0.61, -0.27]	40%
Mohlig 2005	-0.48	[-0.67, -0.30]	47%
Tapola (i) 2005	-0.42	[-0.58, -0.26]	32%
Tapola (ii) 2005	-0.43	[-0.60, -0.26]	36%
Tapola (iii) 2005	-0.45	[-0.63, -0.27]	43%
Ulmius 2009	-0.46	[-0.64, -0.27]	45%
Afagh 2011	-0.48	[-0.67, -0.30]	46%
Juvonen (i) 2011	-0.49	[-0.67, -0.31]	43%
Juvonen (ii) 2011	-0.48	[-0.66, -0.29]	47%
Juvonen (iii) 2011	-0.50	[-0.67, -0.32]	41%
Lappi (i) 2013	-0.49	[-0.67, -0.30]	46%
Lappi (ii) 2013	-0.47	[-0.65, -0.28]	47%
Lappi (iii) 2013	-0.43	[-0.60, -0.26]	36%
Lappi (i) 2014	-0.49	[-0.67, -0.31]	45%
Lappi (ii) 2014	-0.46	[-0.65, -0.28]	47%
Hartvigsen (i) 2014a	-0.47	[-0.66, -0.28]	47%
Hartvigsen (ii) 2014a	-0.46	[-0.64, -0.28]	46%
Hartvigsen (i) 2014b	-0.47	[-0.66, -0.28]	47%
Hartvigsen (ii) 2014b	-0.48	[-0.66, -0.29]	47%
Hartvigsen (iii) 2014b	-0.49	[-0.67, -0.30]	46%
Giulia 2016	-0.47	[-0.66, -0.28]	47%
Ullah (i) 2022	-0.48	[-0.67, -0.30]	47%
Ullah (ii) 2022	-0.49	[-0.67, -0.31]	44%
Ponzo 2024	-0.48	[-0.66, -0.29]	47%
Xu (i) 2024	-0.48	[-0.67, -0.29]	47%
Xu (ii) 2024	-0.48	[-0.66, -0.29]	47%
Aberg 2024	-0.48	[-0.66, -0.29]	47%
Moreira 2024	-0.48	[-0.67, -0.29]	47%

Table S12. Sensitivity test of postprandial glucose iPeak in clinical studies

Study omitted	SMD	95% CI	I^2 (%)
Overall	-0.52	[-0.80; -0.25]	63%
Hamberg 1989	-0.54	[-0.82, -0.25]	68%
Cherbut 1994	-0.54	[-0.82, -0.26]	68%
Juntunen (i) 2003	-0.55	[-0.83, -0.26]	67%
Juntunen (ii) 2003	-0.55	[-0.83, -0.26]	68%
Juntunen (iii) 2003	-0.53	[-0.82, -0.24]	68%
Tapola (i) 2005	-0.43	[-0.65, -0.21]	48%
Tapola (ii) 2005	-0.44	[-0.67, -0.21]	52%
Tapola (iii) 2005	-0.47	[-0.72, -0.21]	61%
Ulmius 2009	-0.50	[-0.78, -0.22]	67%
Lappi (i) 2010	-0.54	[-0.83, -0.25]	68%
Lappi (ii) 2010	-0.54	[-0.83, -0.25]	68%
Lappi (iii) 2010	-0.52	[-0.80, -0.23]	68%
Juvonen (i) 2011	-0.56	[-0.84, -0.28]	65%
Juvonen (ii) 2011	-0.53	[-0.82, -0.24]	68%
Juvonen (iii) 2011	-0.56	[-0.84, -0.27]	66%
Brennan (i) 2012	-0.56	[-0.83, -0.29]	65%
Brennan (ii) 2012	-0.51	[-0.79, -0.22]	68%
Stefan 2018	-0.53	[-0.82, -0.24]	68%
Ullah (i) 2022	-0.54	[-0.83, -0.25]	68%
Ullah (ii) 2022	-0.55	[-0.84, -0.27]	66%
Xu (i) 2024	-0.53	[-0.82, -0.24]	68%
Xu (ii) 2024	-0.53	[-0.82, -0.24]	68%
Moreira 2024	-0.51	[-0.79, -0.22]	67%

Table S13. Sensitivity test of postprandial insulin Peak in clinical studies

Study omitted	SMD	95% CI	I^2 (%)
Overall	-0.29	[-0.44; -0.15]	0%
Hamberg 1989	-0.29	[-0.44, -0.14]	0%
Lu (i) 2000	-0.30	[-0.45, -0.15]	0%
Lu (ii) 2000	-0.29	[-0.44, -0.14]	0%
Mohlig 2005	-0.31	[-0.46, -0.16]	0%
Ulmius 2009	-0.30	[-0.45, -0.15]	0%
Juvonen (i) 2011	-0.32	[-0.47, -0.17]	0%
Juvonen (ii) 2011	-0.29	[-0.44, -0.14]	0%
Juvonen (iii) 2011	-0.31	[-0.47, -0.16]	0%
Lappi (i) 2013	-0.27	[-0.42, -0.12]	0%
Lappi (ii) 2013	-0.29	[-0.44, -0.13]	0%
Lappi (iii) 2013	-0.29	[-0.44, -0.14]	0%
Lappi (i) 2014	-0.29	[-0.44, -0.14]	0%
Lappi (ii) 2014	-0.30	[-0.45, -0.15]	0%
Hartvigsen (i) 2014a	-0.31	[-0.46, -0.16]	0%
Hartvigsen (ii) 2014a	-0.27	[-0.42, -0.12]	0%
Hartvigsen (i) 2014b	-0.30	[-0.45, -0.15]	0%
Hartvigsen (ii) 2014b	-0.28	[-0.43, -0.13]	0%
Hartvigsen (iii) 2014b	-0.28	[-0.43, -0.13]	0%
Ullah (i) 2022	-0.26	[-0.41, -0.11]	0%
Ullah (ii) 2022	-0.29	[-0.44, -0.13]	0%
Ponzo 2024	-0.29	[-0.44, -0.14]	0%
Xu (i) 2024	-0.31	[-0.46, -0.16]	0%
Xu (ii) 2024	-0.31	[-0.46, -0.16]	0%

Table S14. Sensitivity test of postprandial insulin iPeak in clinical studies

Study omitted	SMD	95% CI	I^2 (%)
Overall	-0.24	[-0.41; -0.06]	0%
Hamberg 1989	-0.24	[-0.42, -0.06]	5%
Cherbut 1994	-0.23	[-0.40, -0.05]	3%
Lia 1997	-0.24	[-0.42, -0.06]	5%
Juntunen (i) 2003	-0.19	[-0.37, -0.01]	0%
Juntunen (ii) 2003	-0.20	[-0.38, -0.02]	0%
Juntunen (iii) 2003	-0.21	[-0.39, -0.03]	0%
Ulmius 2009	-0.25	[-0.43, -0.06]	6%
Lappi (iii) 2010	-0.25	[-0.43, -0.07]	5%
Lappi (ii) 2010	-0.25	[-0.44, -0.07]	4%
Lappi (i) 2010	-0.23	[-0.42, -0.05]	6%
Juvonen (i) 2011	-0.28	[-0.46, -0.10]	0%
Juvonen (ii) 2011	-0.23	[-0.41, -0.04]	7%
Juvonen (iii) 2011	-0.27	[-0.45, -0.09]	0%
Stefan 2018	-0.24	[-0.42, -0.05]	7%
Ullah (i) 2022	-0.24	[-0.42, -0.05]	7%
Ullah (ii) 2022	-0.24	[-0.43, -0.05]	8%
Xu (i) 2024	-0.26	[-0.44, -0.08]	1%
Xu (ii) 2024	-0.26	[-0.44, -0.08]	3%

Table S15. Sensitivity test of fasting glucose in preclinical studies

Study omitted	SMD	95% CI	I^2 (%)
Overall	-1.18	[-1.56; -0.80]	82%
Ohara (i) 2000	-1.14	[-1.50, -0.77]	98%
Ohara (ii) 2000	-1.16	[-1.52, -0.80]	98%
Laerke 2008	-1.16	[-1.52, -0.81]	98%
Kim 2010	-1.10	[-1.46, -0.75]	98%
Neyrinck 2012	-1.15	[-1.51, -0.79]	98%
Hartvigsen (i) 2013	-1.12	[-1.48, -0.76]	98%
Hartvigsen (ii) 2013	-1.12	[-1.48, -0.76]	98%
Hartvigsen (iii) 2013	-1.08	[-1.42, -0.73]	98%
Agyekum 2015	-1.16	[-1.52, -0.80]	98%
Abulnaja (i) 2015	-1.15	[-1.51, -0.79]	98%
Abulnaja (ii) 2015	-1.15	[-1.51, -0.79]	98%
Alauddin (i) 2016	-1.12	[-1.48, -0.76]	98%
Alauddin (ii) 2016	-1.12	[-1.48, -0.76]	98%
Kieffer 2016	-1.17	[-1.52, -0.81]	98%
Nie (i) 2018	-1.15	[-1.51, -0.79]	98%
Nie (ii) 2018	-1.08	[-1.43, -0.74]	98%
Nie (iii) 2018	-1.12	[-1.48, -0.76]	98%
Nie (iv) 2018	-1.12	[-1.48, -0.76]	98%
Sarma (i) 2018	-1.17	[-1.52, -0.82]	98%
Sarma (ii) 2018	-1.17	[-1.52, -0.82]	98%
Neyrinck 2018	-1.12	[-1.48, -0.76]	98%
Li (i) 2019	-1.13	[-1.49, -0.77]	98%
Li (ii) 2019	-1.11	[-1.46, -0.75]	98%
Yang (i) 2019	-1.13	[-1.49, -0.76]	98%
Yang (ii) 2019	-1.14	[-1.51, -0.78]	98%
Yang (iii) 2019	-1.14	[-1.50, -0.77]	98%
Chen 2020	-1.13	[-1.49, -0.77]	98%
Ai (i) 2021	-1.15	[-1.51, -0.79]	98%
Ai (ii) 2021	-1.09	[-1.45, -0.74]	98%
Ai (iii) 2021	-1.06	[-1.39, -0.72]	98%
Nie 2021	-1.12	[-1.49, -0.76]	98%
Alzahrani (i) 2022	-1.14	[-1.50, -0.78]	98%
Alzahrani (ii) 2022	-1.08	[-1.43, -0.73]	98%
Alzahrani (iii) 2022	-1.09	[-1.44, -0.74]	98%
Alzahrani (iv) 2022	-1.09	[-1.44, -0.74]	98%
Alzahrani (v) 2022	-1.08	[-1.42, -0.74]	98%
Alzahrani (vi) 2022	-1.09	[-1.43, -0.74]	98%

An (i) 2022	-1.16	[-1.52, -0.81]	98%
An (ii) 2022	-1.14	[-1.51, -0.78]	98%
An (iii) 2022	-1.16	[-1.52, -0.81]	98%
An (iv) 2022	-1.15	[-1.51, -0.79]	98%
Abdou 2023	-1.15	[-1.51, -0.79]	98%
Fang 2024	-1.13	[-1.50, -0.77]	98%
Agista (i) 2024	-1.15	[-1.51, -0.79]	98%
Agista (ii) 2024	-1.13	[-1.50, -0.77]	98%

Table S16. Sensitivity test of fasting insulin in preclinical studies

Study omitted	SMD	95% CI	I^2 (%)
Overall	-1.07	[-1.92; -0.23]	84%
Ohara (i) 2000	-0.99	[-1.83, -0.16]	98%
Laerke 2008	-0.96	[-1.79, -0.12]	98%
Kim 2010	-1.08	[-1.86, -0.29]	98%
Neyrinck 2012	-0.95	[-1.78, -0.12]	98%
Hartvigsen (i) 2013	-1.07	[-1.86, -0.29]	98%
Hartvigsen (ii) 2013	-1.04	[-1.86, -0.23]	98%
Hartvigsen (iii) 2013	-1.07	[-1.86, -0.29]	98%
Nielsen 2015	-1.00	[-1.84, -0.17]	98%
Zhang 2016	-0.98	[-1.81, -0.14]	98%
Alauddin (i) 2016	-0.96	[-1.80, -0.13]	98%
Alauddin (ii) 2016	-0.89	[-1.71, -0.08]	98%
Kieffer 2016	-1.00	[-1.83, -0.17]	98%
Nie (i) 2018	-1.00	[-1.83, -0.16]	98%
Nie (ii) 2018	-0.87	[-1.68, -0.07]	98%
Nie (iii) 2018	-0.85	[-1.65, -0.06]	98%
Nie (iv) 2018	-0.93	[-1.77, -0.10]	98%
Sarma (i) 2018	-1.00	[-1.83, -0.17]	98%
Sarma (ii) 2018	-0.84	[-1.63, -0.05]	98%
Sarma (iii) 2018	-0.84	[-1.63, -0.06]	98%
Neyrinck 2018	-0.97	[-1.80, -0.13]	98%
Li (ii) 2019	-0.97	[-1.80, -0.13]	98%
Nie 2021	-0.97	[-1.80, -0.13]	98%
Alzahrani (i) 2022	-0.99	[-1.82, -0.15]	98%
Alzahrani (ii) 2022	-0.99	[-1.82, -0.15]	98%
Alzahrani (iii) 2022	-0.86	[-1.65, -0.06]	98%
Alzahrani (iv) 2022	-0.84	[-1.63, -0.05]	98%
Alzahrani (v) 2022	-0.87	[-1.68, -0.06]	98%
Alzahrani (vi) 2022	-0.84	[-1.63, -0.06]	98%
Abdou (i) 2023	-1.01	[-1.84, -0.17]	98%
Abdou (ii) 2023	-1.07	[-1.85, -0.30]	98%
Agista (i) 2024	-0.98	[-1.82, -0.15]	98%
Agista (ii) 2024	-0.96	[-1.79, -0.12]	98%

Table S17. Sensitivity Test of HbA1c in preclinical studies

Study omitted	SMD	95% CI	I^2 (%)
Overall	-2.93	[-5.48; -0.38]	94%
Hartvigsen (i) 2013	-2.33	[-5.20, 0.54]	98%
Hartvigsen (ii) 2013	-2.80	[-6.08, 0.47]	98%
Hartvigsen (iii) 2013	-2.16	[-4.74, 0.43]	98%
Abulnaja (i) 2015	-3.67	[-6.40, -0.95]	97%
Abulnaja (ii) 2015	-3.67	[-6.40, -0.95]	97%

Table S18. Sensitivity test of HOMA-IR in preclinical studies

Study omitted	SMD	95% CI	I^2 (%)
Overall	-2.44	[-3.66; -1.22]	86%
Mio 2002	-2.01	[-2.95, -1.07]	96%
Neyrinck 2012	-1.97	[-2.96, -0.97]	96%
Nielsen 2015	-1.99	[-2.84, -1.14]	95%
Alauddin (i) 2016	-1.98	[-2.97, -1.00]	96%
Alauddin (ii) 2016	-1.85	[-2.82, -0.88]	97%
Neyrinck 2018	-1.97	[-2.96, -0.98]	96%
Li (ii) 2019	-1.99	[-2.97, -1.02]	96%
Nie 2021	-1.67	[-2.55, -0.80]	95%
Alzahrani (i) 2022	-1.99	[-2.97, -1.00]	96%
Alzahrani (ii) 2022	-1.61	[-2.43, -0.80]	95%
Alzahrani (iii) 2022	-1.68	[-2.51, -0.85]	96%
Alzahrani (iv) 2022	-1.63	[-2.46, -0.80]	95%
Alzahrani (v) 2022	-1.66	[-2.48, -0.84]	95%
Alzahrani (vi) 2022	-1.71	[-2.56, -0.87]	96%
Agista (i) 2024	-1.99	[-2.97, -1.01]	96%
Agista (ii) 2024	-1.95	[-2.95, -0.96]	96%

Table S19. Sensitivity test of fasting glucose in clinical studies

Study omitted	SMD	95% CI	I^2 (%)
Overall	-0.10	[-0.16; -0.03]	61%
Anderson 1984	-0.10	[-0.17, -0.04]	42%
Vaaler 1986	-0.10	[-0.16, -0.03]	42%
Jenkins 2002	-0.09	[-0.16, -0.03]	41%
Juntunen 2003	-0.11	[-0.17, -0.05]	28%
McIntosh (i) 2003	-0.09	[-0.15, -0.03]	35%
McIntosh (ii) 2003	-0.09	[-0.15, -0.03]	39%
Lu 2004	-0.08	[-0.13, -0.02]	27%
Garcia 2007	-0.09	[-0.16, -0.03]	41%
Ulmius 2009	-0.10	[-0.17, -0.03]	43%
Cheng 2010	-0.09	[-0.16, -0.03]	40%
Maki (i) 2012	-0.09	[-0.16, -0.02]	39%
Maki (ii) 2012	-0.10	[-0.17, -0.02]	44%
Raimondi 2016	-0.09	[-0.16, -0.02]	42%
Aoe 2018	-0.11	[-0.18, -0.04]	46%
Schioldan 2018	-0.10	[-0.17, -0.03]	47%
Salden (i) 2018	-0.09	[-0.16, -0.03]	43%
Salden (ii) 2018	-0.10	[-0.16, -0.03]	44%
Muller 2020	-0.11	[-0.19, -0.04]	44%
Xue 2020	-0.11	[-0.17, -0.04]	37%
Liu 2021	-0.10	[-0.17, -0.03]	48%
Saphyakhajorn 2022	-0.10	[-0.18, -0.03]	47%
Schmidt-Combest 2023	-0.10	[-0.17, -0.03]	46%
Ghorbani 2024	-0.09	[-0.15, -0.03]	38%

Table S20. Sensitivity test of fasting insulin in clinical studies

Study omitted	SMD	95% CI	I^2 (%)
Overall	-0.02	[-5.89; -5.93]	63%
Juntunen 2003	-0.23	[-6.79, 6.33]	76%
Lu 2004	0.99	[-4.9, 6.88]	72%
Garcia 2007	-0.59	[-6.54, 5.36]	74%
Ulmius 2009	0.60	[-5.92, 7.13]	74%
Cheng 2010	-1.51	[-6.56, 3.54]	62%
Maki (i) 2012	0.94	[-5.3, 7.19]	72%
Maki (ii) 2012	0.44	[-6.09, 6.98]	76%
Raimondi 2016	0.42	[-6.2, 7.04]	74%
Schioldan 2018	1.21	[-4.18, 6.61]	68%
Salden (i) 2018	0.18	[-5.75, 6.1]	75%
Salden (ii) 2018	-0.70	[-6.83, 5.44]	74%
Xue 2020	-0.70	[-7.05, 5.65]	74%
Liu 2021	-1.07	[-6.86, 4.73]	71%
Saphyakhajorn 2022	-0.10	[-6.7, 6.51]	76%
Schmidt-Combest 2023	0.31	[-5.95, 6.58]	76%

Table S21. Sensitivity test of HbA1c in clinical studies

Study omitted	SMD	95% CI	I^2 (%)
Overall	-0.01	[-0.03; 0.01]	39%
Vaaler 1986	-0.01	[-0.03, 0.01]	5%
Jenkins 2002	-0.01	[-0.03, 0.01]	2%
Cheng 2010	-0.01	[-0.03, 0.01]	7%
Aoe 2018	-0.01	[-0.04, 0.01]	8%
Xue 2020	0.00	[-0.03, 0.02]	10%
Liu 2021	-0.01	[-0.04, 0.01]	27%
Saphyakhajorn 2022	0.00	[-0.03, 0.02]	53%

Table S22. Sensitivity test of HOMA-IR in clinical studies

Study omitted	SMD	95% CI	I^2 (%)
Overall	-0.02	[-0.35; 0.31]	67%
Cheng 2010	-0.07	[-0.42, 0.28]	71%
Raimondi 2016	0.12	[-0.20, 0.44]	38%
Schioldan 2018	0.03	[-0.35, 0.41]	74%
Xue 2020	-0.18	[-0.44, 0.08]	31%
Saphyakhajorn 2022	0.01	[-0.43, 0.45]	71%

Study	D1	D2	D3	D4
Ohara (2000)	!	+	+	!
Lærke (2008)	!	-	+	+
Kim (2010)	+	+	+	+
Neyrinck (2012)	!	+	+	+
Hartvigsen (2013)	+	+	+	+
Alauddin (2016)	!	+	+	+
Abulnaja (2015)	+	!	+	+
Agyekum (2015)	+	+	+	+
Nielsen (2015)	!	+	+	+
Kieffer (2016)	+	+	+	+
Zhang (2016)	+	+	!	!
Neyrinck (2018)	!	+	-	!
Nie (2018)	+	+	+	+
Sarma (2018)	+	-	+	+
Li (2020)	+	+	+	+
Yang (2019)	+	+	+	+
Chen (2020)	+	+	+	+
Ai (2021)	+	+	+	+
Nie (2021)	!	!	+	+
Alzahrani (2022)	!	+	+	+
An (2022)	+	+	+	!
Mio (2022)	+	+	+	!
Abdou (2023)	!	+	+	+
Fang (2024)	+	+	+	+
Agista (2024)	!	+	+	!

Overall

+ Low risk
! Some concerns
- High risk

D1: Randomisation process

D2: Missing outcome data

D3: Selection of the reported result

D4: Bias from other sources

Figure S1. Risk of bias assessment of preclinical studies

Study	D1	DS	D2	D3	D4	D5
Hamberg (1989)	!	!	!	+	+	+
Cherbut (1994)	!	+	+	+	+	+
Lia (1997)	+	+	+	+	+	+
Lu (2000)	+	+	+	+	+	+
Juntunen (2003)	!	+	+	+	+	+
Möhlig (2005)	!	+	+	+	+	+
Tapola (2005)	!	+	+	+	+	+
Hlebowicz (2009)	!	+	+	+	+	+
Ulmius (2009)	+	+	+	+	+	+
Lappi (2010)	!	+	+	+	+	+
Afaghi (2011)	!	+	+	+	+	+
Juvonen (2011)	!	+	+	+	+	+
Brennan (2012)	!	+	+	+	+	+
Lappi (2013)	!	+	+	+	+	+
Hartvigsen (2014)	+	+	+	+	+	+
Hartvigsen (2014)	+	+	+	+	+	+
Lappi (2014)	!	+	+	+	+	+
Lafond (2015)	+	+	+	+	+	+
Giulia (2016)	+	+	+	+	+	+
Lee (2016)	!	+	+	+	+	+
Shi (2017)	!	+	+	+	+	+
Camps (2018)	!	+	+	+	+	+
Ullah (2022)	+	+	+	+	+	+
Åberg (2025)	!	+	+	+	+	+
Ponzo (2024)	+	+	+	+	+	+
Moreira (2024)	!	+	+	+	+	+

\+ Low risk

! Some concerns

\- High risk

D1:Randomisation process

DS: Bias arising from period and carryover effects

D2: Deviations from the intended interventions

D3: Missing outcome data

D4: Measurement of the outcome

D5: Selection of the reported result

Xu (2024)	+	+	+	+	+	+
Anderson (1984)	!	NA	+	+	+	+
Vaaler (1985)	!	+	+	+	+	!
Jenkins (2002)	!	-	+	+	+	!
Juntunen (2003)	!	+	+	+	+	+
McIntosh (2003)	!	!	+	+	+	!
Lu (2004)	+	-	+	+	+	!
Garcia (2007)	+	+	+	+	!	+
Cheng (2010)	+	NA	+	+	+	!
Maki (2012)	+	!	+	+	+	+
Raimondi (2016)	+	NA	+	+	+	+
Aoe (2018)	+	NA	+	+	+	+
Salden (2018)	+	NA	+	+	+	!
Schioldan (2018)	-	+	+	!	!	+
Müller (2020)	+	NA	+	+	+	+
Xue (2020)	!	NA	+	+	!	+
Liu (2020)	!	NA	+	-	!	-
Schmidt-Combest (2023)	+	NA	+	+	!	+
Saphyakhajorn (2022)	!	NA	+	+	+	+
Ghorbani (2025)	-	NA	+	+	!	+

Figure S2. Risk of bias assessment of clinical studies

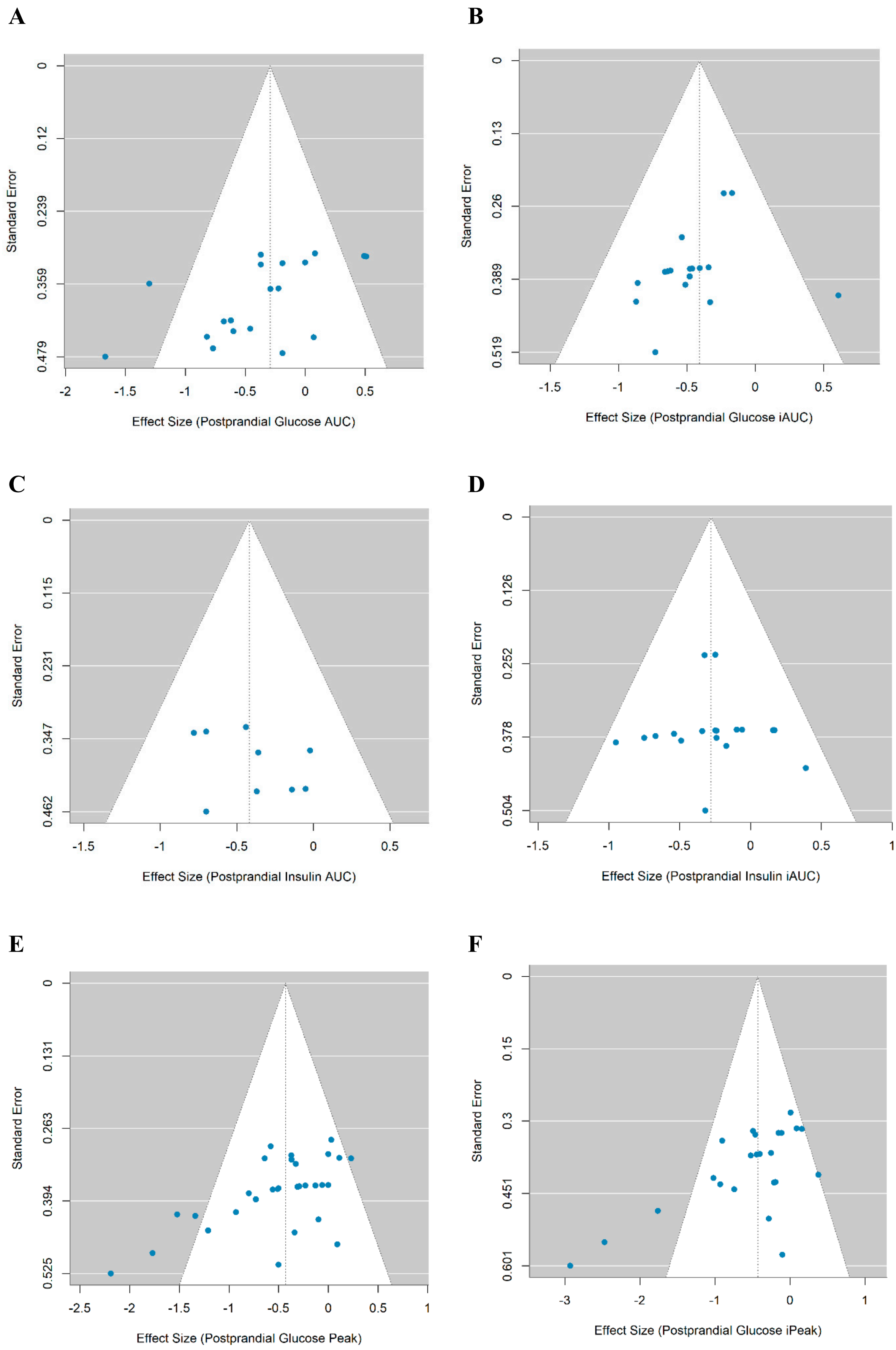
A
Standard Error
Effect Size (Postprandial Glucose AUC)
B
Standard Error
Effect Size (Postprandial Glucose iAUC)
C
Standard Error
Effect Size (Postprandial Insulin AUC)
D
Standard Error
Effect Size (Postprandial Insulin iAUC)
E
Standard Error
Effect Size (Postprandial Glucose Peak)
F
Standard Error
Effect Size (Postprandial Glucose iPeak)

G

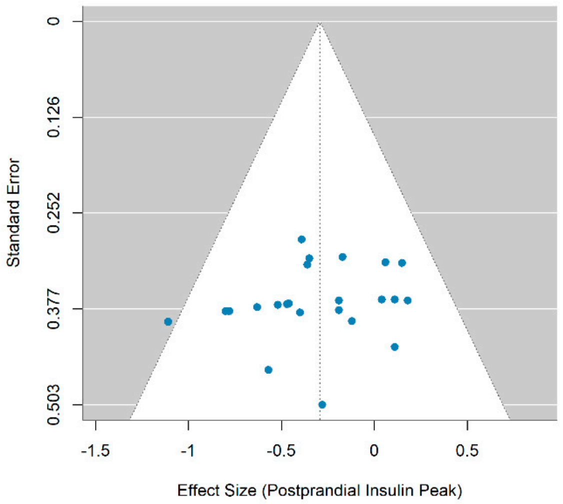

H

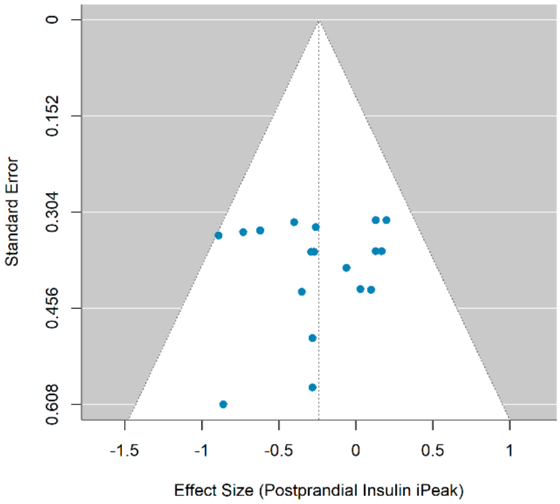

Figure S3. Funnel plot of **(A)** Postprandial glucose AUC (Egger's test P = 0.0038); **(B)** Postprandial glucose iAUC (Egger's test P = 0.2091); **(C)** Postprandial insulin AUC (Egger's test P = 0.4348); **(D)** Postprandial insulin iAUC (Egger's test P = 0.9361); **(E)** Postprandial glucose Peak (Egger's test P < 0.0001); **(F)** Postprandial glucose iPeak (Egger's test P < 0.0001); **(G)** Postprandial insulin Peak (Egger's test P = 0.3545); **(H)** Postprandial insulin iPeak (Egger's test P = 0.7940)

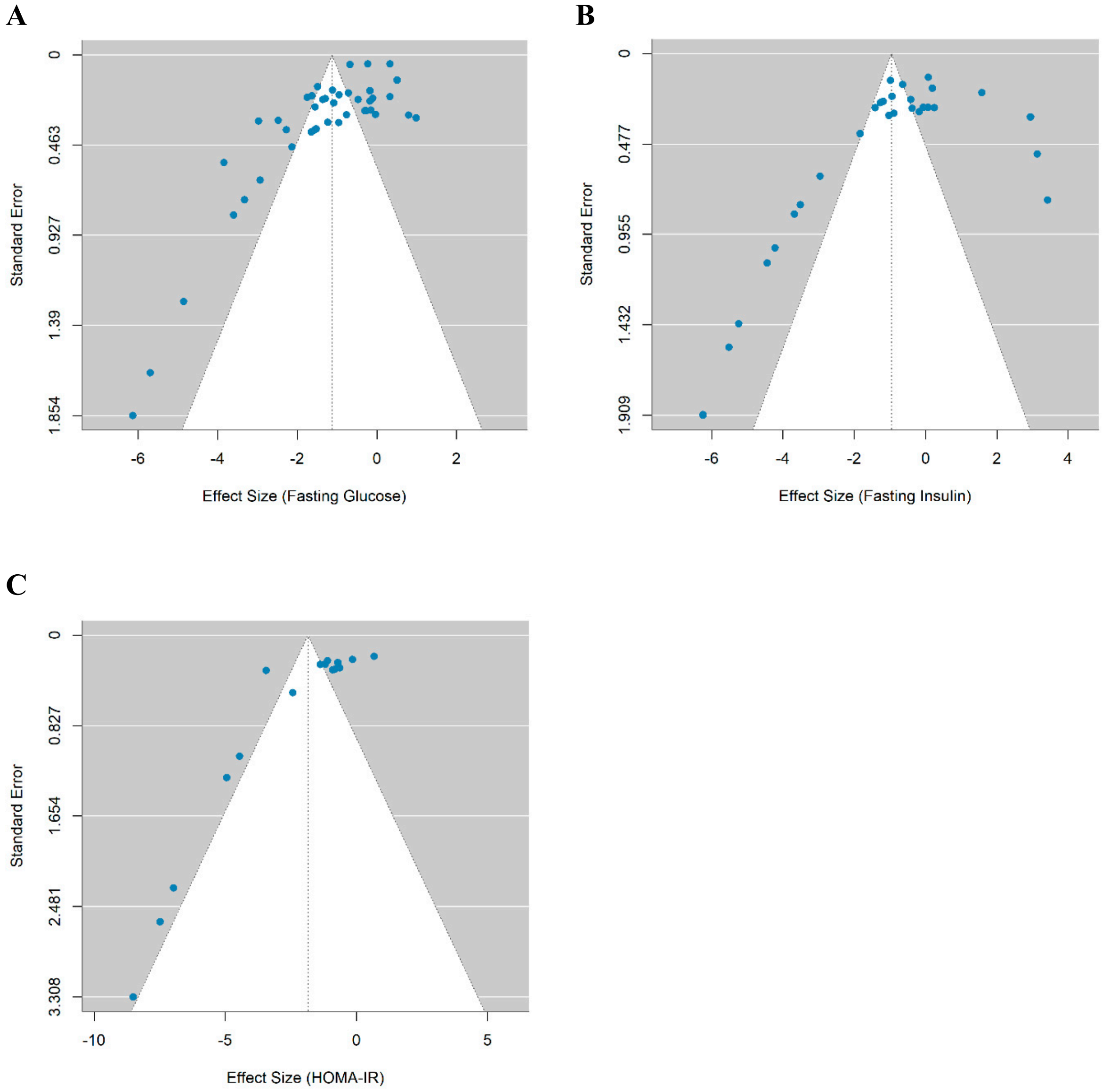

Figure S4. Funnel plot of **(A)** Fasting glucose (Egger's test P < .0001); **(B)** Fasting insulin (Egger's test P < .0001); **(C)** HOMA-IR (Egger's test P < .0001) in preclinical studies.

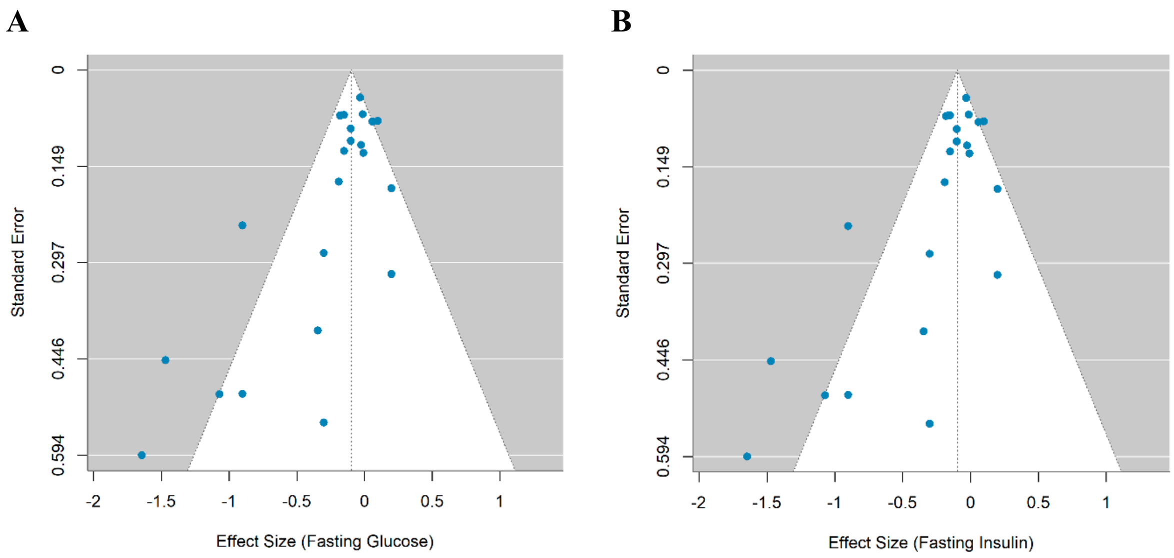

Figure S5. Funnel plot of **(A)** Fasting glucose (Egger's test P < .0001); **(B)** Fasting insulin (Egger's test P < .0001) in clinical studies
